# A systematic review and meta‐analysis of predictive and prognostic models for outcome prediction using positron emission tomography radiomics in head and neck squamous cell carcinoma patients

**DOI:** 10.1002/cam4.6278

**Published:** 2023-06-24

**Authors:** Mahima Merin Philip, Andy Welch, Fergus McKiddie, Mintu Nath

**Affiliations:** ^1^ Institute of Applied Health Sciences, University of Aberdeen Aberdeen UK; ^2^ Institute of Education in Healthcare and Medical Sciences, University of Aberdeen Aberdeen UK; ^3^ National Health Service Grampian Aberdeen UK

**Keywords:** head and neck squamous cell carcinoma, positron emission tomography, prognosis, radiomics, systematic review

## Abstract

**Background:**

Positron emission tomography (PET) images of head and neck squamous cell carcinoma (HNSCC) patients can assess the functional and biochemical processes at cellular levels. Therefore, PET radiomics‐based prediction and prognostic models have the potentials to understand tumour heterogeneity and assist clinicians with diagnosis, prognosis and management of the disease. We conducted a systematic review of published modelling information to evaluate the usefulness of PET radiomics in the prediction and prognosis of HNSCC patients.

**Methods:**

We searched bibliographic databases (MEDLINE, Embase, Web of Science) from 2010 to 2021 and considered 31 studies with pre‐defined inclusion criteria. We followed the CHARMS checklist for data extraction and performed quality assessment using the PROBAST tool. We conducted a meta‐analysis to estimate the accuracy of the prediction and prognostic models using the diagnostic odds ratio (DOR) and average *C*‐statistic, respectively.

**Results:**

Manual segmentation method followed by 40% of the maximum standardised uptake value (SUV_max_) thresholding is a commonly used approach. The area under the receiver operating curves of externally validated prediction models ranged between 0.60–0.87, 0.65–0.86 and 0.62–0.75 for overall survival, distant metastasis and recurrence, respectively. Most studies highlighted an overall high risk of bias (outcome definition, statistical methodologies and external validation of models) and high unclear concern in terms of applicability. The meta‐analysis showed the estimated pooled DOR of 6.75 (95% CI: 4.45, 10.23) for prediction models and the *C*‐statistic of 0.71 (95% CI: 0.67, 0.74) for prognostic models.

**Conclusions:**

Both prediction and prognostic models using clinical variables and PET radiomics demonstrated reliable accuracy for detecting adverse outcomes in HNSCC, suggesting the prospect of PET radiomics in clinical settings for diagnosis, prognosis and management of HNSCC patients. Future studies of prediction and prognostic models should emphasise the quality of reporting, external model validation, generalisability to real clinical scenarios and enhanced reproducibility of results.

## Graphical abstract

We conducted a systematic review and meta‐analysis to assess the significance of positron emission tomography (PET) radiomic features for outcome prediction in head and neck squamous cell carcinoma patients. PET radiomics demonstrated potential to use in clinical settings; however, we recommend that future studies should emphasise the quality of reporting, external model validation, generalisability to real clinical scenarios and enhanced reproducibility of results.

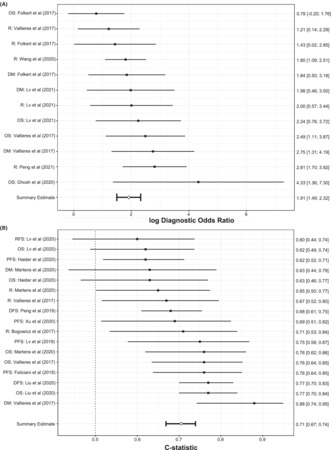

## 1 INTRODUCTION

Head and neck squamous cell carcinoma (HNSCC) is the sixth most common malignancy globally.^1^ HNSCC constitutes a diverse group of cancers originating from the mucosal epithelium of the oral cavity, pharynx, sinonasal tract and larynx.^2^ Despite advances in evaluation and treatment, HNSCC outcomes marginally improved over the past decades attributed to delayed diagnosis and recurrence.^3^ Understanding tumour heterogeneity is key in cancer management as it has implications on tumour development, therapeutic outcomes and survival.^4^

Non‐invasive medical imaging techniques such as magnetic resonance, computed tomography (CT) and positron emission tomography (PET) provide information about tumours.^4^ Tumours exhibiting high intratumoral heterogeneity have been found to have a less favourable prognosis, which may be due to either inherent aggressive characteristics or treatment resistance.^5^ PET outperforms other imaging modalities as an ideal tool for characterising the tumour biology at the macroscopic level^5^, ^6^. PET with 2‐deoxy‐2‐[fluorine‐18]fluoro‐d‐glucose (18F‐FDG), which is a glucose analogue and has similar metabolism as glucose, provides valuable functional information based on increased glucose uptake and glycolytic activity of cancer cells. Hence, PET provides information about functional and biochemical changes in bodily tissues that precede anatomical changes.^7^, ^8^ Clinical implications of PET are already evident in brain tumour, thyroid cancer, non‐small cell lung cancer, breast cancer, oesophageal cancer, pancreatic cancer, colorectal cancer, cervical cancer, sarcoma and lymphoma in addition to head and neck cancer.^9^ PET in head and neck cancer is useful for clinical staging, identifying deep‐seated tumours, detection of unknown primary, distant metastasis, recurrent tumour, nodal staging of locally advanced head and neck cancer, detection of primary tumours, radiotherapy planning and treatment response evaluation.^10^, ^11^, ^12^, ^13^ Standard uptake value (SUV), metabolic tumour volume (MTV) or total lesion glycolysis (TLG) provides information useful for diagnosis, earlier evaluation and treatment response evaluation.^4^ PET radiomic features are found to be better than SUV parameters in some types of cancer in survival outcome prediction.^6^ Textural analysis has been widespread in PET since the late 2000s.^14^ Due to the functional nature and close link to tumour biology, the radiomic features extracted from PET images have the potential to capture the phenotypic differences across the tumours correlated with the stage and prognosis of the disease.^14^

Machine learning models can be trained to recognise patterns in complex PET radiomics data, assisting clinicians with risk assessment, diagnosis and prognosis, thus improving patient care.^15^ By the middle of 2020 among the published radiomics studies only 16% were based on PET or PET/CT.^16^ Owing to the valuable information provided by PET images about tumour heterogeneity, it is essential to perform a systematic review evaluating the current status and potentials of PET radiomic feature‐based models in HNSCC outcomes to direct the course of future research in diagnosis, prognosis and management of HNSCC.

In this study, we present a systematic review to assess the current status of prediction and prognostic models based on pretreatment PET images in HNSCC studies. The objectives of the systematic review are to evaluate the implemented segmentation methods, identify essential radiomic feature‐based predictors, assess model development strategies and estimate the overall performance using meta‐analysis.

## 2 MATERIALS AND METHODS

This review is conducted according to the guidance of preferred reporting items for systematic reviews and meta‐analyses (PRISMA)^17^ and the critical appraisal and data extraction for systematic reviews of prediction modelling studies (CHARMS).^18^ The protocol for the study was registered on the international prospective register of systematic reviews (PROSPERO 2021 Registration number CRD42021287832).^19^

### 2.1 Eligibility criteria

#### 2.1.1 Outcomes of interest

The outcomes of interest and their definitions are as follows:

Overall survival (OS)/All‐cause mortality (ACM): The time from diagnosis to death from any cause or last date of follow‐up.
Recurrence (R): The time between the end of treatment to local or locoregional recurrence of disease progression or death from any cause or last date of follow‐up. We considered disease‐free survival (DFS), relapse‐free survival and recurrence‐free survival (RFS) as recurrence.^20^
Progression‐free survival (PFS): The time from the end of primary treatment to the date of disease progression, death or last follow‐up.
Distant metastasis (DM): The time to first clinical or pathological evidence of disease spread to distant organs or lymph nodes.
Disease‐specific survival (DSS): The time from diagnosis to time to death due to HNSCC.

#### 2.1.2 Inclusion criteria

Studies were included if they met the following criteria:

1. Patients diagnosed with HNSCC cancer pathologically (including anatomic subtypes)
2. FDG‐PET/CT or FDG‐PET scan is done before treatment
3. Radiomic features considered
4. The clinical outcomes of interest were OS/ACM, R, DM, PFS and DSS.
5. Patients are treated with chemotherapy/radiotherapy/surgery/brachytherapy or a combination of these.
6. Studies with a minimum follow‐up time of 1 year were included, and there was no restriction on the last follow‐up time at which the outcome was measured.
7. Provided details of predictive or prognostic models used along with their performance measures.

#### 2.1.3 Exclusion criteria

Studies were excluded if they (a) were based on animal/genomic studies; (b) were comment letters, conference abstracts, meeting abstracts, book chapters, early access, editorial materials, systematic reviews or case reports; (c) had fewer than 50 patients; (d) were MRI or CT studies; (e) studies evaluating treatment response as an outcome; (f) studies not published in English. The details on search strategy, study selection, data extraction and bias assessment are provided in Supporting Information (Tables S1–S3).

We categorised the included studies into prediction models (binary outcome) and prognostic models (time‐to‐event). The prediction model considers binary outcome; that is the outcome can take only one of two values, such as treatment failure or success, or mortality (dead or alive).^21^ Hence, the prediction model is a binary classification problem which is usually assessed by performance metrics, the area under the receiver operating curve (AUC) accuracy, sensitivity and specificity.^22^ The time‐to‐event analysis is used to analyse the time to disease remission, progression or death for cohorts of patients when the time to event is either recorded or censored.^23^ The prognostic modelling approach using the time‐to‐event data is essential when the time between exposure and event is of clinical interest for upgrading treatments and techniques to improve the quality and longevity of life.^24^, ^25^, ^26^ Kaplan–Meier plots, log‐rank test and the Cox proportional hazard model are the commonly used techniques for analysing the time‐to‐event data.^25^, ^27^ The common performance metric used for the prognostic model is the concordance index or *C*‐statistic.^28^

### 2.2 Meta‐analysis

We performed the random effect meta‐analysis to evaluate the overall performance metrics of both predictive models (binary outcome) and prognostic models (time‐to‐event outcome) accounting for the heterogeneity between studies. For the meta‐analysis, we considered all outcomes (OS, recurrence, DSS, DM and PFS) within each predictive and prognostic modelling framework. We performed additional meta‐analyses to compare the performance metrics between manual and threshold‐based segmentation methods for studies involving prediction and prognostic models. Further details are presented in Supporting Information.

## 3 RESULTS

### 3.1 Study selection

**FIGURE 1:**
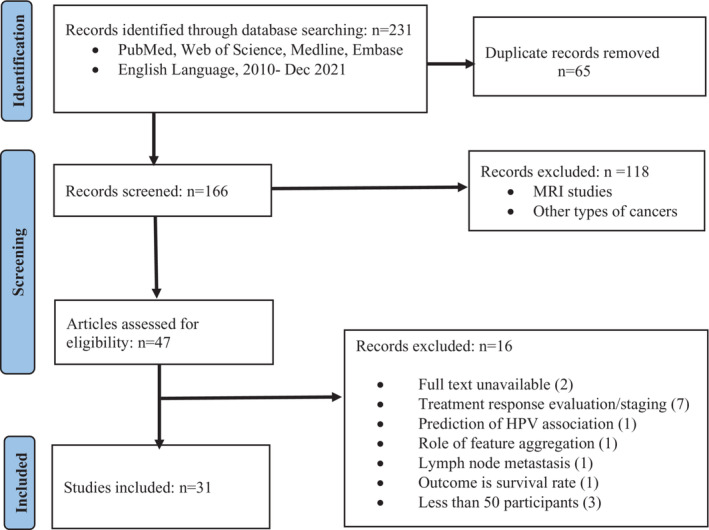
Flow diagram highlighting search strategy and selection of studies.

The initial database search revealed 231 published papers between 2010 and 2021 December. The study selection process resulted in 31 model development studies eligible for the systematic review (Figure 1). The included studies in the review represented about 4500 HNSCC patients, including its subtypes. The main findings of this study are summarised in Tables 1 and 2.

**TABLE 1:** Summary of included studies that reported prediction models of binary outcomes.

Author; year	Segmentation method	Outcome	Model	Performance	Important features
Folkert et al. (2017)^49^	42% SUV_max_	ACM, DM, LF	Multiparametric LR	Internal validation ACM: AUC = 0.65, Sen = 0.67, Spe = 0.60 R: AUC = 0.73, Sen = 0.65, Spe = 0.85 DM: AUC = 0.66, Sen = 0.62, Spe = 0.66 External validation ACM: AUC = 0.60, Sen = 0.58, Spe = 0.62 R: AUC = 0.68, Sen = 0.67, Spe = 0.70 DM: AUC = 0.65, Sen = 0.64, Spe = 0.80	DM‐KPS, T stage, N stage R‐KPS OS‐KPS, T stage
Ghosh et al. (2020)^35^	40% SUV_max_	OS	GBDT	Bal.acc = 88.25%, Sen = 96.5%, Spe = 80%, Precision = 93%, F1 score = 94%, AUC = 0.99	Primary tumour site, MTV, GLCM_Correlation_
Lv et al. (2021)^48^	Manual	OS, DM, R	Ensemble LR	External validation R: AUC = 0.75, Sen = 0.60, Spe = 0.84 DM: AUC = 0.8; Sen = 0.82, Spe = 0.66 OS: AUC = 0.87; Sen = 0.88; Spe = 0.61	No information
Peng et al. (2021)^43^	Manual	R	SVM	AUC = 0.82, Spe = 0.77, Sen = 0.84	Compactness1, GLCM_Entropy_, GLSZM_LZLGE_, NGTDM_Strength_, GLGLM _SGE_
Vallières et al. (2017)^42^	Manual	OS, DM, R	R‐RF, DM‐RF, OS‐RF	External validation R: AUC = 0.69, Sen = 0.63, Spe = 0.68, acc = 0.67 DM: AUC = 0.86, Sen = 0.76, Spe = 0.76, acc = 0.77 OS: AUC = 0.78, Sen = 0.92, Spe = 0.57, acc = 0.65	DM: H&N type, N stage age, tumour volume R‐Age, GLSZM_GLN_, H&N type, N stage, T stage OS‐H&N type, T stage, age, tumour volume, HPV
Wang et al. (2020)^47^	Manual	R	MC	External validation AUC = 0.62, Sen = 0.62, Spe = 0.61, Acc = 0.61	No information
Xie et al. (2020)^36^	Manual	OS, DFS	HNC cohort‐DFS and OS‐RF NPC Cohort DFS‐RF, OS‐SVM	Internal validation HNC Cohort‐DFS: AUC = 0.72 OS: AUC = 0.84 Internal validation NPC Cohort DFS: AUC = 0.70 OS: AUC = 0.82	No information
Zhong et al. (2021)^44^	SUV >1.5 times liver SUV_mean_	PFS	RF	Internal validation AUC = 0.94. Acc = 0.80, Sen = 1, Spe = 0.67, PPV = 1, F1 score = 0.77	MTV, SUVmin, GLZLM_SZLGE_, kurtosis

Abbreviations: Acc, accuracy; ACM, all‐cause mortality; AUC, area under the ROC curve; DFS, disease‐free survival; DM, distant metastasis; GBDT, gradient‐boosted decision tree; GLCM, grey level co‐occurrence matrix; GLGLM, grey level gap length matrix; GLN, grey level non‐uniformity; GLRLM‐grey level run length matrix; GLSZM, grey level size zone matrix; HNC, head and neck cancer; KPS, Karnofsky Performance Status; LF, local failure; LGZE, low grey level zone emphasis; LR, logistic regression; LRHGE, long run high grey level emphasis; LZLGE, large zone low grey‐level emphasis; MC, multi‐classifier; MTV, metabolic tumour volume; NGTDM, Neighbourhood grey tone difference matrix; NPC, nasopharyngeal carcinoma; OS, overall survival; PFS, progression‐free survival; PPV, positive predictive value; R, recurrence; RF, random forest; Sen, sensitivity; SGE, short gap emphasis; Spe, specificity; SUV, standardised uptake value; SZLGE, small zone low grey‐level emphasis; SVM, support vector machine; ZSN, zone size non‐uniformity; ZSV, zone size variance.

**TABLE 2:** Summary of included studies that reported prognostic models of time‐to‐event outcomes.

Author; year	Segmentation method	Outcome	Model	Performance	Important features
Beichel et al. (2019)^54^	Semi‐automatic graph based	DFS	CoxPH	No information	DFS: MTV, glycolysis Q2, Rim average
Bogowicz et al. (2017)^46^	PET‐gradient‐based auto segmentation	R	CoxPH	Internal validation CI_PET_ = 0.74 External validation CI_PET_ = 0.71	R: spherical disproportion, GLSZM_SZLGE_
Chan et al. (2017)^53^	Manual	OS, RFS	CoxPH	No information	OS: age, EBV DNA load, uniformity RFS: Skewness
Cheng et al. (2020)^32^	40% SUV_max_	OS, relapse‐free survival	CoxPH	Internal validation CI: OS: 0.83, relapse‐free survival: 0.78	OS: ECOG 2 or N2c−N3, subgroup 3 PET pattern PFS: ECOG 2 or N2c−N3, subgroup 3 PET pattern
Cheng et al. (2013)^40^	SUV threshold of 2.5	PFS, DSS, OS	CoxPH	No information	PFS: age, tumour TLG, NGLCM_uniformity_ DSS: age, tumour TLG, NGLCM_uniformity_ OS: TLG, NGLCM_uniformity_, HPV positivity
Cheng et al. (2015)^39^	SUV 2.5	PFS, DSS	CoxPH	No information	PFS: GLSZM_ZSN_ (16 bins), NGLCM_Uniformity_ DSS: GLSZM_ZSN_ (16 bins), NGLCM_Uniformity_
Feliciani et al. (2018)^33^	40% SUV_max_	PFS, OS	CoxPH	Internal validation CI: PFS: 0.76, OS: NR, R: NR	PFS: Chemotherapy, GLRLM_LILRE_ OS: Gender, age
Folkert et al. (2017)^49^	42% SUV_max_	ACM, DM, R	ACM = CoxPH DM, R = FG model	No information	ACM: KPS, T stage DM: KPS, T stage, N stage R: KPS
Fujima et al. (2018)^38^	2.5 SUV	OS, PFS	CoxPH	No information	PFS: GLCM_Homogeneity_, Sphericity
Ger et al. (2019)^41^	PET‐NR	OS	CoxPH	External validation AUC (PET) = 0.59	
Ghosh et al. (2020)^35^	40% SUV_max_	OS	CoxPH	Internal validation Bal.acc = 81.5%, precision = 90%, TPR = 93%, F1‐score = 91.5%, TNR = 70%	OS: Primary tumour site, MTV, GLCM_Correlation_
Guezennec et al. (2019)^2^	40% SUV_max_	OS, RFS	CoxPH	No information	OS: MTV, GLCM_Correlation_, treatment
Haider et al. (2020)^45^	Manual	PFS, OS	RSF	Internal validation CI: PFS: 0.62 OS: 0.63	No information
Kimura et al. (2021)^71^	30% SUV_max_	DFS, OS	CoxPH	No information	OS: Entropy, dissimilarity DFS: GLCM_Entropy_, GLZLM_LZHGE_, GLRLM_SRE_
Lafata et al. (2021)^29^	Manual	RFS	CoxPH	No information	No information
Lin et al. (2020)^30^	2.5 SUV	OS, PFS	CoxPH	No information	OS: TLG‐M, EBV‐DNA titers PFS: SUV_max_ (M)
Liu et al. (2020)^34^	40% SUV_max_	OS, DFS	CoxPH	Internal validation CI: OS: 0.77, DFS: 0.77	R: NGLDM_Coarseness_, SMTV OS: NGLDM_Coarseness_, SMTV
Lv et al. (2019)^52^	Manual	PFS	CoxPH	Internal CI: 0.75	PFS: M stage, Age, VCA‐IgA, N stage, PET‐SUVmid_HLH (wavelet based)
Lv et al. (2020)^50^	Manual	RFS, OS	CoxPH	External validation CI: RFS: 0.60, OS: 0.64	RFS: Age, GLSZM_SZHGE_, GLRLM_LRHGE_, B3 OS: NR
Martens et al. (2020)^3^	50% iso‐contour of SUV_peak_	R, DM, OS	CoxPH	External validation CI: R: 0.65, DM: 0.63, OS: 0.76	R: HPV‐status, SUV_mean_, SUV_peak_, histogram gradient, long run low grey‐level emphasis, volume‐difference, coarseness, and grey level non‐uniformity and histogram variation coefficient DM: MATV OS: HPV‐status, SUV_mean_, SUV_max_, least axis length, non‐uniformity, high‐dependence of high grey‐levels, aspherity, major axis length, inversed‐compactness and inversed‐flatness
Oh et al. (2015)^70^	Manual	DFS, OS	CoxPH	No information	OS: NGTDM_Coarsness_ DFS: NGTDM_Coarsness_, NGTDM_Busyness_
Peng et al. (2019)^31^	Manual	DFS, OS	CoxPH	Internal validation CI: DFS: 0.68	No information
Vallières et al. (2017)^42^	Manual	OS, DM, R	RSF	External validation CI: R: 0.67, DM: 0.88, OS: 0.76	DM: H&N type, N stage, age, tumour volume OS: H&N type, T stage, Age, Tumour volume, HPV R: Age, H&N type, N stage, T stage
Wong et al. (2019)^37^	SUV threshold of 2.5	RFS, OS	CoxPH	No information	RFS: transfer constant (Ktrans), TLG, NGLCM_entropy_ OS: Ktrans, blood plasma volume (Vp), SUV, NGLCM _entropy_
Xu et al. (2020)^72^	Manual	PFS	CoxPH	Internal validation CI 0.69	PFS: AJCC stage III‐IV, NGTDM_Complexity_, PET‐GLGLM_SGLGE_
Yoon et al. (2021)^51^	Nestle's adaptive thresholding	DFS, OS	CoxPH	No information	OS: GLZLM_GLNU_ DFS: GLZLM_GLNU_

Abbreviations: ACM, all‐cause mortality; AUC, area under the ROC curve; Bal.Acc, balanced accuracy; CI, concordance index; CoxPH, Cox proportional hazard; DFS, disease‐free survival; DM, distant metastasis; DSS, disease‐specific survival; EBV, Epstein–Barr Virus; ECOG, Eastern Cooperative Oncology Group; FG model, Fine and Gray's proportional sub‐hazards models; GLCM, grey level co‐occurrence matrix; GLGLM, grey level gap length matrix; GLN, grey level non‐uniformity; GLNU, grey‐level non‐uniformity; GLRLM, grey level run length matrix; GLSZM, grey level size zone matrix; GLZLM, grey level zone length matrix; HPV, human papillomavirus; KPS, Karnofsky Performance Status; LGZE, low grey level zone emphasis; LILRE, low‐intensity long‐run emphasis; LRE, long run emphasis; LRHGE, long run high grey‐level emphasis; LZHGE, long zone high grey‐level emphasis; MTV, metabolic tumour volume; NGLCM, normalised grey level co‐occurrence matrix; NGLDM, neighbouring grey‐level dependence matrix; NGTDM, neighbourhood grey tone difference matrix; NR, not reported; OS, overall survival; PFS, progression‐free survival; R, recurrence; RFS, recurrence‐free survival; RSF, random survival forest; SGLGE, short gap low grey‐level emphasis; SZHGE, short zone high grey‐level emphasis; SMTV, standardised metabolic tumour volume; SRE, short run emphasis; SUV, standardised uptake value; SZLGE, small zone low grey‐level emphasis; TLG, total lesion glycolysis; TNR, true negative rate; TPR, true positive rate; VCA‐IgA, EBV capsid Antigen immunoglobulin A; ZSN, zone size non‐uniformity; ZSV, zone size variance.

### 3.2 Study design and patient characteristics

All selected studies were retrospective cohort studies except the study by Lafata et al.^29^ The sample size varied across the studies between 52^30^ and 707,^31^ and 17 of the studies recruited more than 100 patients. The mean age of the patient was ≥50 years in 10 studies. Among the selected studies, 15 considered all disease stages, and 25 studies included T and N grade of the tumour. Chemoradiotherapy is the most frequently mentioned treatment modality (*n* = 26) which was often combined with other treatment strategies like radiotherapy (*n* = 20), surgery (*n* = 6) and biotherapy. The Supporting Information provides further details of sample design and study characteristics (Table S4).

### 3.3 Segmentation methods

PET‐CT was the preferred imaging modality in 30 studies, and in one study, PET images alone were considered.^29^ The manual method was the preferred segmentation method in about 40% of the studies, followed by the fixed percentage of standardised uptake value (SUV) threshold (40% of SUV_max_)^2^, ^32^, ^33^, ^34^, ^35^, ^36^ and fixed value of SUV (2.5 SUV).^30^, ^37^, ^38^, ^39^, ^40^ Other segmentation methods were 42% SUV_max_, 30% SUV_max_, SUV >1.5 times liver SUV_mean_, 50% iso‐contour of SUV_peak_, gradient‐based auto‐segmentation, Nestle's adaptive thresholding, 50% of SUV peak and graph‐based methods. One study did not report the employed segmentation method.^41^ Tables 1 and 2 present a summary of segmentation methods used by included studies.

### 3.4 Types of features

In addition to demographic, pathological and clinical variables, a few studies considered other variables like smoking status, alcohol consumption, treatment/dose, family history of cancer, and body mass index. For radiomic features, 19 studies reported histogram‐based features and 13 discussed shape‐based features. Grey level co‐occurrence matrix (GLCM), grey level run length matrix (GLRLM), grey level size zone matrix/ grey level zone length matrix (GLSZM/GLZLM) and neighbourhood grey tone difference matrix (NGTDM) features are essential second‐order features. The number of features for the model development considered varied between 14 and 6294, with 15 studies incorporating more than 50 features.

### 3.5 Feature engineering, feature selection and dimensionality reduction

We noted that 29 studies did not report the handling of the outliers and missing values. Some studies identified the skewness of the data and addressed it by the logarithmic transformation^42^ or incorporating median values.^42^, ^43^ The min–max scalar was employed by one study.^35^ Only a single study^44^ reported dummy variables for handling categorical data. About 23 studies examined feature selection strategies. These included model‐based feature selection like forward selection or backward elimination, least absolute shrinkage and selection operator (LASSO) based embedded methods,^31^, ^33^, ^34^, ^41^, ^43^ and ridge regularisation.^3^, ^45^ The principal component analysis^46^ and factor analysis^3^ were two common dimension reduction methods reported. Lafata et al.^29^ implemented an unsupervised clustering algorithm for feature selection.

Few studies^43^, ^44^, ^49^ discussed hyperparameter optimisation using cross‐validation. Three studies^35^, ^36^, ^47^ implemented the synthetic minority oversampling technique (SMOTE), while two others employed standard oversampling method^36^, ^42^ to deal with class imbalances. Almost half of the studies did not discuss the resampling method used. K‐fold cross‐validation and bootstrap resampling were the popular techniques utilised in the remaining studies.

### 3.6 Prediction model with a binary outcome

Table 1 provides the performance metrics of included studies based on internal validation. AUC, sensitivity and specificity were the commonly cited performance metrics. The external validation was performed only in four studies^42^, ^47^, ^48^, ^49^ for OS, DM and recurrence outcomes. Predictive models that reported the best performances were: ensemble logistic regression (OS; AUC = 0.87); random forest model (DM; AUC = 0.86) and ensemble logistic regression (recurrence; AUC = 0.75). The ranges of performance metrics of externally validated models were: OS (AUC = 0.60–0.87, sensitivity = 0.58–0.92 and specificity = 0.57–0.62); DM (AUC = 0.65–0.86, sensitivity = 0.64–0.82 and specificity = 0.66–0.80); recurrence (AUC = 0.62–0.75, sensitivity = 0.60–0.67 and specificity = 0.61–0.84).

### 3.7 Prognostic model with a time‐to‐event outcome

CoxPH model was the commonly used prognostic model for the time‐to‐event (survival) data, and the C‐statistic was the preferred performance metric (Table 2). The CoxPH models reported the best performance for recurrence, PFS and OS with *C* indices of 0.78,^32^ 0.76^33^ and 0.83,^32^ respectively. Models were externally validated by five studies.^3^, ^41^, ^42^, ^46^, ^50^, ^51^ For OS prognosis, the random survival forest model^42^ exhibited a similar performance as that of the CoxPH model (*C*‐index = 0.76). The random survival forest model documented the best performance for DM prognosis with a *C*‐index of 0.88.^42^

### 3.8 Important radiomic and non‐radiomic features

Table 1 highlights the important features associated with the prediction model. T stage^42^, ^49^ and tumour volume^35^, ^42^ were significant non‐radiomic predictors for OS. However, except GLCM_correlation,_ ^35^ no unique radiomic feature was consistently identified across the studies for OS prediction. For DM, non‐radiomic features such as age, N stage, T stage, tumour volume and Karnofsky Performance Status (KPS), and head and neck (H&N) type were important.^42^, ^49^ KPS, shape‐based compactness, age, H&N type, N stage, T stage were critical non‐radiomic features for recurrence.^42^, ^43^, ^49^ Second‐order features like NGTDM_Strength_, GLSZM_GLN_, GLCM_Entropy_, GLSZM_LZLGE_ and GLGLM_SGE_ (grey level gap length matrix) features were reported as the key radiomic features.^42^, ^43^ The features predictive of PFS were MTV, SUV_min_ and the radiomic feature GLSZM_SZLGE_ and histogram‐kurtosis.^44^

Table 2 presents significant features reported for the prognostic models. For the prediction of OS, important predictors were age,^33^, ^42^, ^53^ T stage,^42^, ^49^ primary tumour site,^35^, ^42^ EBV DNA,^30^, ^53^ and HPV status.^40^, ^42^ Other crucial features included PET quantitative features like MTV,^2^, ^34^, ^35^ SUV,^3^, ^37^ TLG,^30^ GLCM‐based features^2^, ^35^ and shape‐based features.^3^ For PFS, first‐order feature uniformity^39^, ^40^ and age^40^, ^52^ were significant features. For recurrence prediction, age,^42^, ^50^ tumour volume^34^, ^54^ and second‐order radiomic features were predominant features. For DSS, NGLCM_uniformity_ was a key feature,^39^, ^40^ while for DM, clinical variables and N stage were crucial for prognostic models.^42^, ^49^

### 3.9 Risk of bias in the studies

**FIGURE 2:**
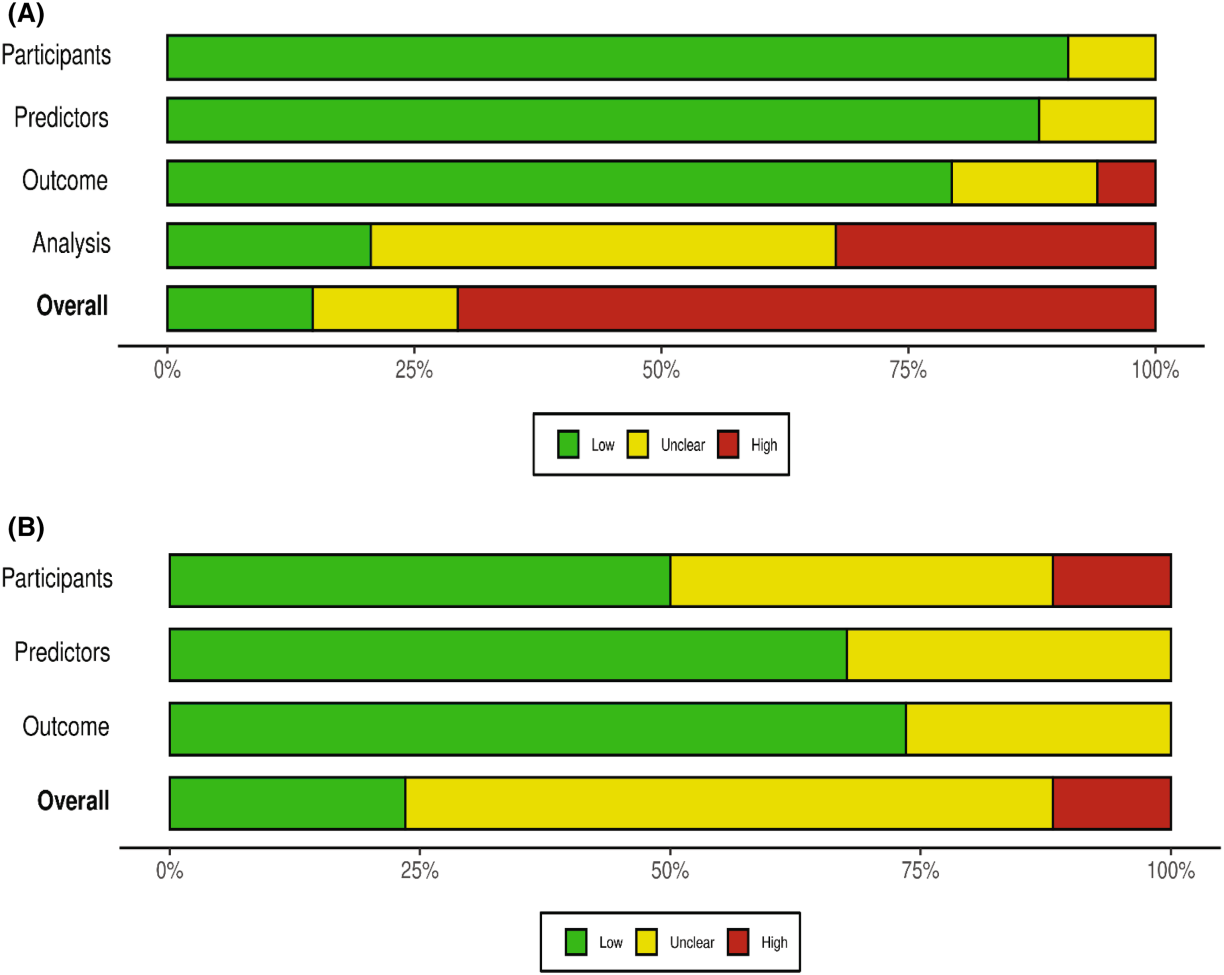
Quality assessment using PROBAST for (A) the overall risk of bias at participants, predictors, outcome and analysis levels and the overall pooled data; (B) the overall applicability of the included studies at participants, predictors and outcome levels and the overall pooled data.^55^

We assessed the quality of the included studies using PROBAST. The assessment of ROB and applicability is presented in Figure 2, and additional details are provided in Supporting Information (Table S6; Figure S1). The overall ROB was low or unclear in 10 studies and high in 21 studies. Within ROB, high bias was observed in the ‘analysis’ domain in 25% of the studies and low bias in the ‘participant’ domain (93.5%). In terms of overall applicability, ROB was of low concern in 8 studies, unclear concern in 22 and high concern in 1 study. The responses to signalling questions in PROBAST are presented in Supporting Information (Table S6).

### 3.10 Meta‐analysis

**FIGURE 3:**
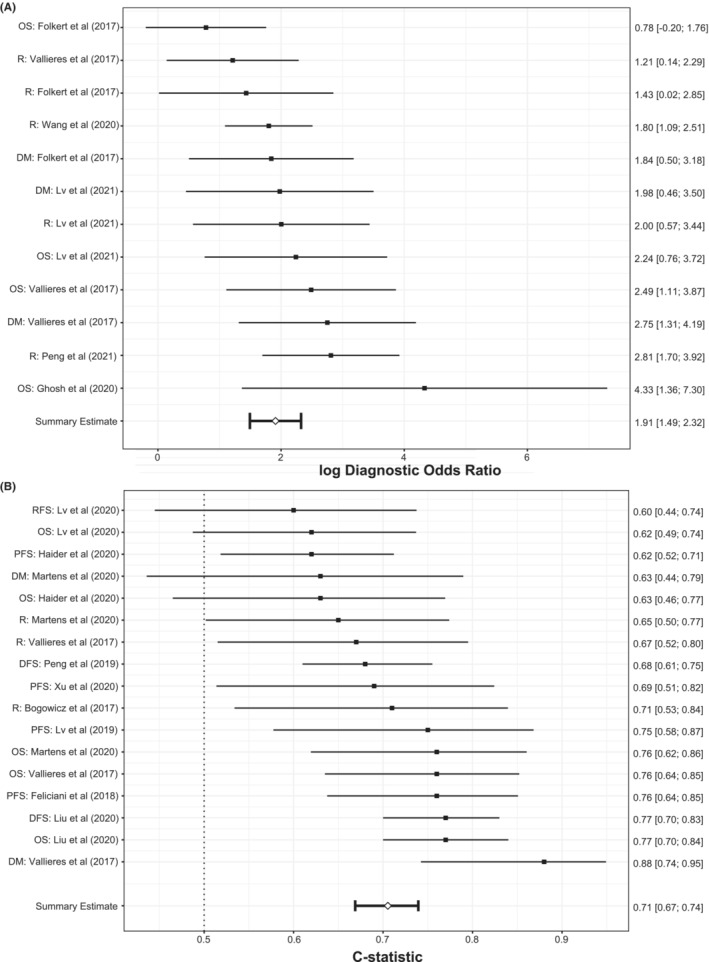
(A) Forest plot of the summary estimate of logarithmic DOR and the corresponding 95% confidence interval (CI) of prediction models (Performance metrics were based on external validation except for Ghosh et al.^35^ and Peng et al.,^43^ where the performance metrics were based on internal validation). (B) Forest plot of pooled *C*‐statistic and the corresponding 95% CI of prognostic models (Performance metrics were based on internal validation except for Bogowicz et al.,^46^ Lv et al.,^50^ Martens et al.^3^ and Vallières et al.,^42^ where the performance metrics were based on external validation).

We conducted a meta‐analysis of performance metrics of both predictive and prognostic models. For predictive models, there was no evidence of heterogeneity between models (*Q* = 10.97 with 11 degrees of freedom, *p* = 0.446). Figure 3A presents the estimates of the logarithm of the diagnostic odds ratio and summary estimate, and the corresponding 95% confidence interval (CI). The estimated log DOR ranged from 0.78 to 4.33, with the pooled estimate being 1.91 (95% CI: 1.49, 2.32). The pooled estimate was equivalent to the estimated DOR of 6.75 (95% CI: 4.45, 10.23), suggesting that PET‐features‐based models showed good predictive performance. We have also conducted separate meta‐analyses of studies that used manual and SUV‐based segmentation methods. The pooled estimate of DOR of studies that employed the manual method (8.36; 95% CI: 5.47, 12.78) was higher compared to studies that used the threshold‐based segmentation method (4.89; 95% CI: 1.79, 13.38) (Figure S2).

The meta‐analysis of prognostic models did not suggest evidence of heterogeneity between models (*Q* = 24.47 with 16 degrees of freedom, *p* = 0.080). Figure 3B presents the estimated and pooled *C*‐statistic and the 95% CI. The estimated C‐statistic ranged from 0.60 to 0.88, with the pooled estimate being 0.71 (95% CI: 0.67, 0.74), suggesting that the overall performance metric of PET‐based prognostic models was reasonable. A single outcome variable did not exhibit consistently higher (or lower) performance metrics for either prediction or prognostic models among all studies. The estimate of pooled *C*‐statistic was smaller in studies that employed the manual segmentation method (0.68; 95% CI: 0.63, 0.71) compared to studies that used the threshold‐based segmentation method (0.75; 95% CI: 0.70, 0.78) (Figure S3).

## 4 DISCUSSION

Over the past few years, there has been an increase in interest in exploring the relevance of PET‐based radiomics in HNSCC outcome management. PET‐based radiomics enable the quantitative evaluation of tumour heterogeneity, thereby facilitating personalised treatment approaches. Various predictive and prognostic models have been employed to predict adverse outcomes in HNSCC. This systematic review aimed to evaluate the role of pretreatment PET radiomics in predicting adverse outcomes in HNSCC patients.

We systematically reviewed 31 studies to assess the current status of the modelling framework for predicting outcomes in HNSCC patients. We identified important segmentation methods and critical radiomic features predictive of outcome and evaluated the predictive and prognostic performance of the reported models using meta‐analysis.

Manual segmentation was the preferred segmentation method followed by threshold‐based (40% SUV_max_ and 2.5 SUV) methods. The accuracy of manual segmentation is reported to be high, and it is a widely accepted standard if done by an expert radiologist. It is, however, often time‐consuming and operator‐dependent.^56^ Although determining the optimal threshold value is challenging, the recommended threshold values are between 41% and 50% of SUV_max_. The 2.5 absolute SUV method is software and observer‐independent, and easy to use.^57^ As automatic segmentation is an active field of research, automatic segmentation methods are recommended in future studies.^58^

We observed that feature engineering techniques, imbalance class adjustment techniques and hyperparameter tuning were minimally explored in the included studies. Applications of suitable feature selection and feature engineering methods were critical as they impacted the performance of the models.^59^, ^60^, ^61^

Based on externally validated models, the ensemble logistic regression for OS and recurrence prediction and the random forest classifier for DM produced the best performance metrics among prediction models. For other types of cancers, similar performance metrics were reported using different prediction models.^62^, ^63^, ^64^, ^65^

For the time‐to‐event dataset, the survival analysis using the CoxPH model and C‐index as a performance metric was widely used and is a recommended method by several researchers.^66^ The proportional hazard assumption of the Cox survival model is crucial; however, only Cheng et al.^53^ confirmed checking the assumption. If the assumption is not met, modelling approaches should include an appropriate stratified analysis or extend the model by incorporating time‐dependent predictors. The random forest survival model has limited assumptions with a wide range of applications.^67^

Four studies reported external validation of fitted predictive models, and five studies reported external validation of prognostic models, consistent with the recommendation by Nikolas et al.^58^ An exhaustive assessment of model performance metrics was lacking in most studies. The assessment of performance metrics should be externally validated for discrimination (*C*‐statistic, AUC), calibration (calibration in the large, calibration slope) and overall performance (Brier score, scaled Brier score). The validation of the developed model in a new patient set structurally different from the training cohort is necessary for confirming the developed model's generalisability and reproducibility, and wider implementation in clinical practice.^68^

Our result suggests that the overall ROB was higher in more than 60% of studies and is of high concern regarding applicability in a single study. The high ROB is primarily due to high bias in the following areas: handling the missing data, appropriate use of prespecified or standard outcome definition, the number of participants, accounting for the complexities in the data, and evaluation of appropriate model performance measures and lack of external validation.

The meta‐analysis of performance metrics of both predictive and prognostic models demonstrated reasonable performance accuracy. It is important to emphasise that most of these models were not externally validated; therefore, the performance metrics of some models could be overly optimistic. However, considering the direction of these metrics and associated uncertainties, predictive and prognostic models incorporating PET features and other clinical attributes illustrated promising opportunities for further development and refinement of these models toward clinical application.

### 4.1 Limitations and recommendations

The current systematic review has some limitations. Most studies are retrospective cohorts with varying sample sizes. These differences limit comparison in terms of predictive features and the robustness of the model. Key comparisons of model development and validation are also limited as the majority of the studies did not report detailed methodologies. Despite the fact that PET‐based models demonstrated satisfactory performance, the literature suggests that combining PET and CT‐based features might improve model performance in head and neck cancer prognosis.^41^, ^42^, ^52^ The review and evaluation of models incorporating CT radiomics were outside the scope of the current systematic review.

We recommend more prospective studies with larger sample sizes focussing on different imaging modalities, including PET and CT‐based radiomics, and the underlying mechanism of tumour heterogeneity. All studies should report outliers, acknowledge missing values handlings and provide a detailed account of the implemented pipeline (like feature selection, dimensionality reduction, techniques to address the class imbalance, hyperparameter tuning, model development, and internal and external validation) for enhanced reproducibility. Studies should incorporate appropriate performance metrics adhering to prediction and prognostic model reporting tools like CONSORT, STROBE and STARD.^69^

## 5 CONCLUSION

The systematic review explored the current status of existing prediction and prognostic models using clinical variables and PET radiomics in managing HNSCC. Both prediction and prognostic models demonstrated reliable diagnostic accuracy for detecting adverse outcomes, suggesting the prospect of using PET radiomics in clinical settings for diagnosis, prognosis and management of HNSCC patients. Future studies should emphasise the quality of reporting, external model validation, generalisability to real clinical scenarios and enhanced reproducibility of results.

## AUTHOR CONTRIBUTIONS

**Mahima Merin Philip:** Conceptualization (equal); data curation (lead); formal analysis (lead); investigation (lead); methodology (lead); resources (equal); software (lead); validation (lead); visualization (equal); writing – original draft (equal); writing – review and editing (equal). **Andy Welch:** Conceptualization (supporting); methodology (supporting); project administration (supporting); supervision (supporting); validation (supporting); writing – review and editing (supporting). **Fergus McKiddie:** Conceptualization (supporting); methodology (supporting); project administration (supporting); resources (supporting); supervision (supporting); validation (supporting); writing – review and editing (supporting). **Mintu Nath:** Conceptualization (equal); data curation (supporting); formal analysis (supporting); funding acquisition (lead); investigation (supporting); methodology (supporting); project administration (lead); resources (equal); software (supporting); supervision (lead); validation (equal); visualization (equal); writing – original draft (equal); writing – review and editing (equal).

## FUNDING INFORMATION

MMP was funded by the University of Aberdeen under the Elphinstone Scholarship. The University of Aberdeen Open Access Fund supported the open access publication.

## CONFLICT OF INTEREST STATEMENT

The authors have no competing interests to declare relevant to this article's content.

## CONSENT TO PUBLISH

All authors provided consent to publish this study.

## Supporting information


Data S1.

## Data Availability

All data generated or analysed during this study are included in this published article and its Supplementary Information files.
