# Supplementary material for: A systematic review and meta‐analysis of predictive and prognostic models for outcome prediction using positron emission tomography radiomics in head and neck squamous cell carcinoma patients
Source: Cancer Med. 2023 Jun 24;12(15):16181–94. doi: 10.1002/cam4.6278 (PMC10469753; doi:10.1002/cam4.6278)
Supplement: Supplementary file 1 — Data S1. [file CAM4-12-16181-s001.pdf]

SUPPLEMENTARY INFORMATION

A systematic review and meta-analysis of predictive and prognostic models for outcome prediction using PET radiomics in Head and Neck Squamous Cell Carcinoma patients

Mahima Merin Philip[1], Andy Welch[2], Fergus McKiddie[3], Mintu Nath[1*]

[1]Institute of Applied Health Sciences, University of Aberdeen, Aberdeen AB25 2ZD, UK

[2]Institute of Education in Healthcare and Medical Sciences, University of Aberdeen, Aberdeen AB25 2ZD, UK

[3]National Health Service Grampian, Aberdeen AB15 6RE, UK

Contents

Abbreviations 2
Methods 4
  Information sources and search strategy 4
  Study selection 4
  Data extraction 4
  Quality assessment of the included studies 5
  Meta-analysis 5
Supplementary Table S1: Medline search 6
Supplementary Table S2: Embase search 9
Supplementary Table S3: Web of science search 12
Supplementary Table S4: Summary of included studies 15
Supplementary Table S5: Summary of models of included studies 23
Supplementary Table S6: PROBAST assessment of Risk of Bias and applicability of included studies. 44
Fig. S1 Quality analysis of the included studies based on PROBAST 46
Fig. S2 Forest plots of prediction models on subset of studies 47
Fig. S3 Forest plots of prognostic models on subset of studies 48
References 49

Abbreviations

**ACM**	All Cause Mortality
**AUC**	Area calculated under the receiver operating characteristic (ROC) curve
**BMI**	Body Mass Index
**CHARMS**	Critical Appraisal and Data Extraction for Systematic Reviews of Prediction Modelling Studies
**C-Index**	Concordance Index
**CoxPH**	Cox Proportional Hazard
**CT**	Computed Tomography
**DA**	Discriminant Analysis
**DFS**	Disease Free Survival
**DM**	Distant Metastasis
**DOR**	Diagnostic Odds Ratio
**DSS**	Disease-Specific Survival
**EBV-DNA**	Epstein-Barr virus- Deoxyribonucleic acid
**FDG-PET/CT**	Fluorodeoxyglucose-positron emission tomography/computed tomography
**GBDT**	Gradient Boosted Decision Tree
**GLCM**	Gray Level Co-occurrence Matrix
**GLGLM**	Gray-Level Gap Length Matrix
**GLN**	Grey-level Nonuniformity
**GLRLM**	Gray-Level Run Length Matrix
**GLSZM/GLZLM**	Gray Level Size Zone Matrix/Gray Level Zone Length Matrix
**H&N**	Head & Neck
**HNSCC**	Head and Neck Squamous Cell Carcinoma
**HPV**	Human papillomavirus
**k-NN**	k Nearest Neighbour
**KPS**	Karnofsky performance status
**LASSO**	Least Absolute Shrinkage and Selection Operator
**LGLZE**	Low Gray-Level Zone Emphasis
**LR**	Logistic Regression
**LZLGE**	Large Zone Low Gray-Level Emphasis
**MRI**	Magnetic Resonance Imaging
**MTV**	Metabolic Tumour Volume
**N**	Number/No
**NGTDM**	Neighbourhood Grey Tone Difference Matrix
**NR**	Not reported
**OS**	Overall Survival
**PET**	Positron Emission Tomography
**PFS**	Progression Free Survival
**PN**	Probably No
**PRISMA**	Preferred Reporting Items for Systematic Reviews and Meta-analyses
**PROBAST**	Prediction model Risk Of Bias ASsessment Tool
**PY**	Probably Yes

**R**	Recurrence
**RF**	Random Forest
**RFS**	Recurrence Free Survival
**ROB**	Risk of Bias
**SGE**	Short Gap Emphasis
**SM**	Supplementary Material
**SMOTE**	Synthetic Minority Over-sampling Technique
**SNR**	Signal to Noise Ratio
**SUV**	Standardised Uptake Value
**SVM**	Support Vector Machine
**SZLGE**	Small Zone Low Gray-Level Emphasis
**TBR**	Tumour to Background Ratio
**TCIA**	The Cancer Imaging Archive
**TLG**	Total Lesion Glycolysis
**TNM**	tumour, node, metastasis
**USA**	United States of America
**Y**	Yes

Methods

Information sources and search strategy

Relevant articles for the review were identified from a search conducted on Medline, Embase and Web of Science for papers published from January 2010 to December 2021. The last search was conducted on 14/01/2022. The following search terms were combined to identify the relevant studies: (Head and Neck cancer) AND (PET/CT) AND (prediction) AND (radiomics). The detailed search strategy is provided in Tables S1, S2 and S3. The search was restricted to studies published in English and studies in humans. The systematic review filter was used to identify prior systematic reviews published addressing a similar research question and also to modify the search strategy to include articles omitted by mistake if any. The search strategy was developed by a staff of the University library who is proficient in systematic reviews and the final strategies were reviewed by experts within our team.

Study selection

Studies identified from the literature search were exported to Mendeley reference management software (1) and duplicates were removed automatically. This was followed by a manual check to identify any undetected duplicates. The title and abstract of the articles were then screened to remove studies that were not adhering to the eligibility criteria. Then the final assessment for eligibility was evaluated by full-text reading by two independent researchers (MMP and MN). Any disagreements between the reviewers were resolved by consensus.

Data extraction

Data from the 31 relevant studies were exported through a standardised form prepared in MS Word based on the recommendations of CHARMS checklist(2). Any measure of adverse events/clinical outcomes, adhering to the inclusion criteria in the case of HNSCC patients was considered for inclusion. In addition to CHARMS checklist information, other study-related information such as authors and the year of publication, the study objective, data sources, participants, study outcome, candidate predictor, sample size, segmentation method utilised, stage of disease, treatment modality, important features, model development and evaluation like missing data handling, resampling methods, imbalance correction, dimensionality reduction, performance metrics are included (Tables S4 and S5). We categorised the modelling approaches broadly into two categories: prediction model that estimates the probability of an outcome using binary response data, and prognostic model that estimates the risk of a specific endpoint using the time to event data.

Quality assessment of the included studies

Two independent reviewers (MMP and MN) assessed the quality of the prediction models in the included studies using the prediction model risk of bias assessment tool (PROBAST) for studies involving both prediction and prognostic models(3). Risk of bias analysis is based on 4 domains (participants, predictors, outcome and analysis) considering 20 signalling questions answered as 'yes'(Y), probably yes (PY), no(N), probably no (PN) or no information (NI). Further, the ROB in each domain is assessed as low, high or unclear. The overall risk of bias assessment is made using '+', '-' or '?' with '+' indicating a low ROB, '-' indicating 'high ROB' and '?' indicating unclear ROB. The applicability analysis is based on 3 domains, (participants, predictors and outcome) rated as low, high or unclear concern. The overall applicability assessment is made using '+', '-'or '?' with '+', '-' or '?' indicating 'low', 'high' and 'unclear' concerns in terms of applicability.

Meta-analysis

We performed the meta-analysis to evaluate the overall performance metrics of both predictive models (binary outcome variable) and prognostic models (time to event outcome variable). For the meta-analysis, we considered all outcomes (overall survival, recurrence, disease-specific survival, distant metastasis, progression-free survival) within each predictive and prognostic modelling framework. For predictive models, we obtained the true positive, true negative, false positive and false negative outcomes from all models, and employed the univariate meta-analysis of diagnostic accuracy using the diagnostic odds ratio (DOR). DOR measures the model's performance as the ratio of the odds of the prediction being positive if the subject has the outcome, relative to the odds of the prediction being positive if the subject does not have the outcome. We applied a random effect model following the approach of DerSimonian and Laird(4). For the prognostic models, we derived the concordance statistic or C-statistic of each study along with the corresponding standard error and 95% confidence interval. Subsequently, we fitted a random effect model using the restricted maximum likelihood method and estimated the average performance of all models and the 95% confidence interval based on the Knapp and Hartung method(5). To compare the performance of prediction and prognostic models on studies employing manual and thresholding-based segmentation methods, we performed additional meta-analyses on subset of studies for each modelling framework(Fig S2 and Fig S3). The meta-analysis for predictive and prognostic models was implemented using the R packages mada (6) and metamisc(7), respectively.

Supplementary Table S1: Medline search

Conducted on 14 January 2022 = 74 papers

#	Query	Number of articles
1	exp "head and neck squamous cell carcinoma"/	8,123
2	HNSCC.tw.	7,815
3	exp "head and neck carcinoma"/ or exp "head and neck squamous cell carcinoma"/ or exp "head and neck cancer"/	330,819
4	laryngeal cancer$.tw.	5,900
5	laryngeal neoplasm$.tw.	329
6	laryngeal squamous cell carcinoma.tw.	1,859
7	nasal cavity cancer$.tw.	25
8	nasal cavity neoplasm$.tw.	12
9	nasal cavity squamous cell carcinoma$.tw.	9
10	paranasal sinus cancer$.tw.	83
11	paranasal sinus neoplasm$.tw.	21
12	paranasal sinus squamous cell carcinoma$.tw.	2
13	nasopharyngeal cancer$.tw.	1,834
14	nasopharyngeal neoplasm$.tw.	54
15	nasopharyngeal squamous cell carcinoma.tw.	32
16	oral cancer$.tw.	11,714
17	oral neoplasm$.tw.	138
18	oral squamous cell carcinoma.tw.	9,119
19	oropharyngeal cancer$.tw.	3,314
20	oropharyngeal neoplasm$.tw.	48
21	oropharyngeal squamous cell carcinoma.tw.	1,733
22	hypopharyngeal cancer$.tw.	1,196
23	hypopharyngeal neoplasm$.tw.	17

24	hypopharyngeal squamous cell carcinoma.tw.	379
25	salivary gland cancer$.tw.	488
26	salivary gland neoplasm$.tw.	795
27	salivary gland squamous cell carcinoma.tw.	3
28	mouth cancer$.tw.	325
29	mouth neoplasm$.tw.	43
30	mouth squamous cell carcinoma.tw.	30
31	Sinonasal squamous cell carcinoma.tw.	120
32	sinonasal cancer$.tw.	234
33	sinonasal neoplasm$.tw.	86
34	pharyngeal cancer$.tw.	890
35	pharyngeal neoplasm$.tw.	33
36	pharyngeal squamous cell carcinoma.tw.	95
37	1 or 2 or 3 or 4 or 5 or 6 or 7 or 8 or 9 or 10 or 11 or 12 or 13 or 14 or 15 or 16 or 17 or 18 or 19 or 20 or 21 or 22 or 23 or 24 or 25 or 26 or 27 or 28 or 29 or 30 or 31 or 32 or 33 or 34 or 35 or 36	334,307
38	exp positron emission tomography/	70,858
39	PET scan.tw.	3,488
40	(PET adj CT).tw.	25,635
41	(positron emission tomography adj computed tomography).tw.	6,414
42	(FDG adj PET).tw.	23,867
43	(fluorodeoxyglucose adj positron emission tomography).tw.	6,157
44	38 or 39 or 40 or 41 or 42 or 43	80,036
45	radiomics.mp.	2,780
46	radiomic$.tw.	2,971
47	texture.tw.	23,692
48	textur* analysis.tw.	2,594
49	45 or 46 or 47 or 48	26,361
50	prediction/ or computer prediction/ or survival prediction/	0
51	exp treatment outcome/ or exp treatment failure/ or exp treatment planning/ or exp treatment response/	1,167,149

52	prediction$.tw.	303,296
53	survival prediction$.tw.	1,621
54	prognosis/ or cancer prognosis/	560,620
55	overall survival.tw.	162,199
56	local recurrence.tw.	28,570
57	distant metastasis.tw.	18,591
58	50 or 51 or 52 or 53 or 54 or 55 or 56 or 57	1,980,735
59	37 and 44 and 49 and 58	75
60	limit 59 to (english language and humans and yr="2010 -Current" and medline)	74
61	limit 60 to yr="2010 - 2021"	74

Supplementary Table S2: Embase search

Conducted on 14 January 2022 = 93 papers

#	Query	Number of articles
1	exp "head and neck squamous cell carcinoma"/	31,673
2	HNSCC.tw.	14,638
3	exp "head and neck carcinoma"/ or exp "head and neck squamous cell carcinoma"/ or exp "head and neck cancer"/	194,235
4	laryngeal cancer$.tw.	7,431
5	laryngeal neoplasm$.tw.	286
6	laryngeal squamous cell carcinoma.tw.	2,508
7	nasal cavity cancer$.tw.	43
8	nasal cavity neoplasm$.tw.	19
9	nasal cavity squamous cell carcinoma$.tw.	12
10	paranasal sinus cancer$.tw.	123
11	paranasal sinus neoplasm$.tw.	23
12	paranasal sinus squamous cell carcinoma$.tw.	7
13	nasopharyngeal cancer$.tw.	2,974
14	nasopharyngeal neoplasm$.tw.	64
15	nasopharyngeal squamous cell carcinoma.tw.	52
16	oral cancer$.tw.	16,796
17	oral neoplasm$.tw.	171
18	oral squamous cell carcinoma.tw.	13,206
19	oropharyngeal cancer$.tw.	5,757
20	oropharyngeal neoplasm$.tw.	58
21	oropharyngeal squamous cell carcinoma.tw.	2,925
22	hypopharyngeal cancer$.tw.	1,757
23	hypopharyngeal neoplasm$.tw.	17

24	hypopharyngeal squamous cell carcinoma.tw.	577
25	salivary gland cancer$.tw.	790
26	salivary gland neoplasm$.tw.	1,226
27	salivary gland squamous cell carcinoma.tw.	5
28	mouth cancer$.tw.	373
29	mouth neoplasm$.tw.	34
30	mouth squamous cell carcinoma.tw.	46
31	Sinonasal squamous cell carcinoma.tw.	206
32	sinonasal cancer$.tw.	362
33	sinonasal neoplasm$.tw.	137
34	pharyngeal cancer$.tw.	1,158
35	pharyngeal neoplasm$.tw.	26
36	pharyngeal squamous cell carcinoma.tw.	159
37	1 or 2 or 3 or 4 or 5 or 6 or 7 or 8 or 9 or 10 or 11 or 12 or 13 or 14 or 15 or 16 or 17 or 18 or 19 or 20 or 21 or 22 or 23 or 24 or 25 or 26 or 27 or 28 or 29 or 30 or 31 or 32 or 33 or 34 or 35 or 36	213,970
38	exp positron emission tomography/	193,652
39	PET scan.tw.	12,168
40	(PET adj CT).tw.	67,141
41	(positron emission tomography adj computed tomography).tw.	11,777
42	(FDG adj PET).tw.	52,530
43	(fluorodeoxyglucose adj positron emission tomography).tw.	9,840
44	38 or 39 or 40 or 41 or 42 or 43	227,161
45	exp radiomics/	4,034
46	radiomic$.tw.	7,026
47	texture.tw.	38,192
48	textur* analysis.tw.	4,489
49	45 or 46 or 47 or 48	44,310
50	prediction/ or computer prediction/ or survival prediction/	447,682
51	exp treatment outcome/ or exp treatment failure/ or exp treatment planning/ or exp treatment response/	2,212,464

52	prediction$.tw.	480,232
53	survival prediction$.tw.	3,112
54	prognosis/ or cancer prognosis/	815,164
55	overall survival.tw.	354,439
56	local recurrence.tw.	49,519
57	distant metastasis.tw.	35,581
58	50 or 51 or 52 or 53 or 54 or 55 or 56 or 57	3,647,831
59	37 and 44 and 49 and 58	172
60	limit 59 to (human and english language and embase and yr="2010 -Current")	93
61	limit 60 to yr="2010 - 2021"	93

Supplementary Table S3: Web of science search

Conducted on 14 January 2022 = 64 papers

	Query	Number of articles
1.	ALL=(HEAD AND NECK CANCER)	75686
2.	ALL=(HEAD AND NECK CARCINOMA)	47539
3.	AB=(HNSCC)	7122
4.	TI=(HNSCC)	1588
5.	ALL=(head and neck squamous cell carcinoma)	32403
6.	(((TI=(nasal cavity neoplasm$)) OR AB=(nasal cavity neoplasm$)) OR TI=(nasal cavity squamous cell carcinoma$)) OR AB=(nasal cavity squamous cell carcinoma$)	453
7.	(TI=(nasal cavity cancer$)) OR AB=(nasal cavity cancer$)	391
8.	(TI=(laryngeal squamous cell carcinoma)) OR AB=(laryngeal squamous cell carcinoma)	2475
9.	(TI=(laryngeal neoplasm$)) OR AB=(laryngeal neoplasm$)	282
10.	(TI=(LARYNGEAL CANCER$)) OR AB=(LARYNGEAL CANCER$)	5012
11.	(((((TI=(oropharyngeal cancer$)) OR AB=(oropharyngeal cancer$)) OR TI=(oropharyngeal neoplasm$)) OR AB=(orophayngeal neoplasm$)) OR AB=(oropharyngeal squamous cell carcinoma)) OR TI=(oropharyngeal squamous cell carcinoma)	6839
12.	(((((TI=(oral cancer$)) OR AB=(oral cancer$)) OR TI=(oral neoplasm$)) OR AB=(oral neoplasm$)) OR AB=(oral squamous cell carcinoma)) OR TI=(oral squamous cell carcinoma)	47101
13.	(((((TI=(paranasal sinus cancer$)) OR AB=(paranasal sinus cancer$)) OR TI=(paranasal sinus neoplasm$)) OR AB=(paranasal sinus neoplasm$)) OR AB=(paranasal sinus squamous cell carcinoma)) OR TI=(paranasal sinus squamous cell carcinoma)	522
14.	(((((TI=(nasopharyngeal cancer$)) OR AB=(nasopharyngeal cancer$)) OR TI=(nasopharyngeal neoplasm$)) OR AB=(nasopharyngeal neoplasm$)) OR AB=(nasopharyngeal squamous cell carcinoma)) OR TI=(nasopharyngeal squamous cell carcinoma)	5010
15.	(((((TI=(sinonasal cancer$)) OR AB=(sinonasal cancer$)) OR TI=(sinonasal neoplasm$)) OR AB=(sinonasal neoplasm$)) OR AB=(sinonasal squamous cell carcinoma)) OR TI=(sinonasal squamous cell carcinoma)	1178
16.	(((((TI=(mouth cancer$)) OR AB=(mouth cancer$)) OR TI=(mouth neoplasm$)) OR AB=(mouth neoplasm$)) OR AB=(mouth squamous cell carcinoma)) OR TI=(mouth squamous cell carcinoma)	2553

17.	(((((TI=(salivary gland cancer$)) OR AB=(salivary gland cancer$)) OR TI=(salivary gland neoplasm$)) OR AB=(salivary gland neoplasm$)) OR AB=(salivary gland squamous cell carcinoma)) OR TI=(salivary gland squamous cell carcinoma)	3500
18.	(((((TI=(hypopharyngeal cancer$)) OR AB=(hypopharyngeal cancer$)) OR TI=(hypopharyngeal neoplasm$)) OR AB=(hypopharyngeal neoplasm$)) OR AB=(hypopharyngeal squamous cell carcinoma)) OR TI=(hypopharyngeal squamous cell carcinoma)	1531
19.	(((((TI=(pharyngeal cancer$)) OR AB=(pharyngeal cancer$)) OR TI=(pharyngeal neoplasm$)) OR AB=(pharyngeal neoplasm$)) OR AB=(pharyngeal squamous cell carcinoma)) OR TI=(pharyngeal squamous cell carcinoma)	1358
20.	#19 OR #18 OR #17 OR #16 OR #15 OR #14 OR #13 OR #12 OR #11 OR #10 OR #9 OR #8 OR #7 OR #6 OR #5 OR #4 OR #3 OR #2 OR #1	128691
21.	ALL=(positron emission tomography)	61,889
22.	(TI=(PET scan)) OR AB=(PET scan)	18,440
23.	(TI=(PET near/1 CT)) OR AB=(PET near/1 CT)	40,011
24.	(AB=(positron emission tomography near/1 computed tomography)) OR TI=(positron emission tomography near/1 computed tomography)	9,681
25.	(TI=(FDG near/1 PET)) OR AB=(FDG near/1 PET)	28,921
26.	(AB=(fluorodeoxyglucose near/1 positron emission tomography)) OR TI=(fluorodeoxyglucose near/1 positron emission tomography)	6,717
27.	#21 OR #22 OR #23 OR #24 OR #25 OR #26	95,950
28.	#21 OR #22 OR #23 OR #24 OR #25 OR #26 and English (Languages)	93,818
29.	#21 OR #22 OR #23 OR #24 OR #25 OR #26 and English (Languages) and Articles or Review Articles (Document Types)	66,527
30.	#21 OR #22 OR #23 OR #24 OR #25 OR #26 and English (Languages) and Articles or Review Articles (Document Types) a**nd** Exclude – Conference Titles	66,020
31.	ALL=(radiomics)	6,192
32.	(TI=(radiomic$)) OR AB=(radiomic$)	6,325
33.	(TI=(texture)) OR AB=(texture)	134,215
34.	(TI=(textur* analysis)) OR AB=(textur* analysis)	41,680
35.	#31 OR #32 OR #33 OR #34	146,372
36.	#31 OR #32 OR #33 OR #34 and English (Languages)	142,704
37.	#31 OR #32 OR #33 OR #34 and English (Languages) and Articles or Review Articles (Document Types)	112,662
38.	#31 OR #32 OR #33 OR #34 and English (Languages) and Articles or Review Articles (Document Types) and Exclude – Conference Titles	111,544
39.	((TI=(prediction)) OR TI=(COMPUTER PREDICTION)) AND TI=(survival prediction)	2,368

40.	(TI=(prediction$)) OR AB=(prediction$)	746,760
41.	(TI=(survival prediction$)) OR AB=(survival prediction$)	20,693
42.	(TS=(prognosis)) OR TS=(CANCER PROGNOSIS)	334,480
43.	(((((TI=(overall survival)) OR AB=(overall survival)) OR TI=(LOCAL RECURRENCE)) OR AB=(LOCAL RECURRENCE)) OR AB=(DISTANT METASTASIS)) OR TI=(DISTANT METASTASIS)	242,118
44.	(((TS=(treatment near/0 outcome)) OR TS=(treatment near/0 failure)) OR TS=(TREATMENT NEAR/0 PLANNING)) OR TS=(TREATMENT NEAR/0 RESPONSE)	134,974
45.	#39 OR #40 OR #41 OR #42 OR #43 OR #44	1,342,203
46.	#39 OR #40 OR #41 OR #42 OR #43 OR #44 and Articles or Review Articles (Document Types) and English (Languages) and Exclude – Conference Titles and Exclude – Book Series Titles	1,108,691
47.	#46 AND #38 AND #30 AND #20	65
48.	#46 AND #38 AND #30 AND #20 and 24TH CONGRESS OF THE EUROPEAN ASSOCIATION FOR CRANIO MAXILLO FACIAL SURGERY (Exclude – Conference Titles)	64

Supplementary Table S4: Summary of included studies

Author; year	Objective	Study design	Data source	Country of study	Patient location	Sample size; M/F	Age	Follow up time	Site	Overall AJCC stage	Tumour grade	Treatment	Imaging modality	Segmentation method
Beichel et al (2019)(8)	Identify promising features	R	Hospital based (2004-2008)	USA	USA?	58, M-47,F-11	Median 55(21-80)Y	median 48.8 (5.4-124.3)m	Tonsil, base of tongue, oro, Naso, Hypo, Pyriform Sinus.	NR	T2,T3, T4,T4a,T4b, N0,N1,N2a, N2b,N2c,N3	CRT	Pre& Post PET/CT	Semi automatic-graph based (on 3D slicer)
Bogowicz et al (2017)(9)	Prediction of local tumour control	R	Hospital based	Switzerland	Switzerland	TC-121 VC-51	Median TC-59 (34-73) VC-58 (47 – 75)Y	Median-TC-64mo VC-22mo	HNSCC (Oro,hypo,larynx, oral cavity)	III,IV	T1,T2,T3,T4,N0,N1,N2,N3	CRT	CT & PET	CT-manual PET-gradient based auto segmentation
Chan et al (2017)(10)	Prediction of OS and Recurrence Free Survival	R	Hospital based (2006-2009)	Taiwan	Taiwan	101 (M-79,F-22)	Mean-50.5 ± 14Y	Median-5.14 years (2-110)m	Naso	I,II,III,IVa,IVb	T1,T2,T3,T4,N0,N1,N2,N3	RT,CRT	PET/CT	Manual Verified-SUV threshold of 2.5
Cheng et al (2013)(11)	Prognostic value of textural features	R	Hospital based (2006-2010)	Taiwan	Taiwan	70(M-66, F-4)	Median-52y.	Median 27(-5.23-74.10)m	Oro	III,IVa,IVb	T3, T4,N0-N2a,N2b-N3	Chemotherapy,biotherapy,RT	PET/CT	SUV threshold of 2.5
Cheng et al (2015)(12)	Predict outcomes and risk	R	Hospital based (2006-2012)	Taiwan	Taiwan	88 , M-82, F-6	Median-51.5Y	32months (range 5 – 92months)	Oro	III,IVa,IVb	T3,T4,N0,N2a,N2b,N3	CRT,bioradiotherapy, RT	PET/CT	SUV 2.5, validation-42% SUVmax &

	stratification													adaptive threshold.
Cheng et al (2020)(13)	Prognostic model for patients with Minor Salivary Gland Carcinoma	R	Hospital based (2007-2016)	Taiwan	Taiwan	75(TC-45,VC-30) M-40,F-35	Median-52(20-81)Y	Median-59.5 (range-2.6-140.9)m	Salivary gland Carcinoma (oral cavity, oro, nasal cavity/paranasal sinus, naso, hypo, larynx, ear)	I,II,III,IV	T1,T2,T3,T4,N0,N2b,N2c,N3	Surgery,RT, CRT	PET/CT	40%SUVmax
Feliciani et al (2018)(14)	Prediction of OS	R	Hospital based(2010-2017)	Italy	Italy	90(M-68,F-22)	Median-60 (range-22-87)Y	Median-38 (24-848)M	HNSCC (oral cavity, oro, hypo, naso, larynx)	III,IV	T1,T2,T3,T4,N0,N1,N2,N3	CRT	PET/CT	40%SUVmax
Folkert et al (2017)(15)	Predict risk of ACM, LF,DM	R	Hospital based (multi-centre TC &VC) TC-2002-2009 VC-2003-2009	USA	TC-USA VC-USA	TC-174; M-152, F-22<br><br>VC-65; M-51, F-14	Mean TC- 57 (27–84) Y VC-58 (38–78)Y	Median TC-55 (6-112mo); VC-28(2–83)mo	Oro(Tonsil, base of tongue, soft palate and posterior pharyngeal wall)	III,IV	T1,T2,T3,T4,N0,N1,N2,N3	CRT	Pre & Post treatment PET/CT	42% SUVmax
Fujima et al (2018)(16)	Prediction of PFS and OS.	R	Hospital based(2009-2013)	Japan	Japan	54(M-46,F-8)	Median 61(range-39-76)Y	Median-progression free gp-49.5m(18-78)m, survivor group -	Pharynx (oro, hypo)	NR	T1,T2,T3,T4a,T4b,N0,N1,N2a,N2b,N2c,N3	CRT	PET/CT	2.5 SUV

								49.8(18-78)m;						
Ger et al (2019) (17)	Comparing imaging protocols and its effect in outcome prediction	R	Hospital based CT-(2004-2013) PET-2004-2013	USA	USA?	CT-377(TC)+349(VC),PET-345(TC)+341(VC)	Median-CT TC-(59(21-87)-VC-57(30-80); PET TC-(60(34-87) VC-58(35-90)	NR	Mostly Oro	I,II,III,IV	T1,T2,T3,T4,N0,N1,N2,N3	RT	PET &CT	CT-Manual PET- NR
Ghosh et al (2020)(18)	Patient stratification	R	TCIA (2003-2013)	India	USA	132; M-113,F-19	Mean 57.27(range -47.5 - 66.9)	At least 5 yrs	HNSCC (oro, naso, hypo, glottis, oral cavity, sinus)	I,II,III,IV	NR	Surgery	PET/CT	40%SUVmax
Guezennec et al (2019)(19)	Assess prognostic value of texture indices	R	Hospital based (2012-2015)	France	France	284 M-245,F-39	median = 63.7 (57.6-69.2)Y	Mean-24.2 ± 15.9 mo	oral cavity, oro, hypo, naso larynx	I,II,III,IV	IS,T1,T2,T3,T4	Surgery, CRT,RT,Chemotherapy	PET/CT	40%SUVmax
Haider et al (2020) (20)	Explore added value of radiomics biomarker in prognostication and risk	R	Hospital based (2009-2019) and TCIA (2003-2013 &2006-2014)	USA	Hospital based-USA TCIA based-USA &Canada	PFS-311(M-253,F-110) OS-306(M-249,F-106),	PFS-mean, (SD)-60.61(9.24)Y OS-60.60(9.28)Y	Median-PFS-1170(798-1645)d, OS-1197(818-1656)d	Oro	I,II,III,IV	T1,T2,T3,T4,N0,N1,N2,N3	CRT,bioradiotherapy,RT,surgery.	PET/CT	Manual

	startification beyond AJCC staging													
Kimura et al (2021)(21)	Assess prognostic value of PET textural features	R	Hospital based (2008-2019)	Japan	Japan	81(M-44,F-37)	Median 67.3y(32-88y	Median-50.1(6.3-133.7)m	Oral SCC (Tongue, Gingiva, Floor of Mouth, Buccal Mucosa)	I,II,III,IV	T1,T2,T3,T4,N0,N1,N2,N3	Surgery	PET/CT	30%SUVmax
Lafata et al (2021) (22)	Prognostic value of intra treatment PET radiomics	PC	Hospital based(2012-2016)	USA	USA	64(M-53,F-11)	Median-59.2±9y	Median-3.9y	Oro	NR	NR	CRT	Pre & intra treatment PET	Manual
Lin et al (2020)(23)	Relationship between PET derviv ed texture parameters and outcome	R	Hospital based(2006-2016)	Taiwan	Taiwan	52(M-44,F-8)	median age=51±13y	Median-17.5m	Naso	NR	T1,T2,T3,T4,N0,N1.N2,N3	CRT	PET/CT	2.5 SUV
Liu et al (2020)(24)	Generating survival model	R	TCIA(2003-2013)	China	USA	Total-171 (TC-115(M-100,F-15) VC-56(47,9))	TC-<60y=71 ≥60y=44. VC-	NR	HNSCC (Oro,larynx, oral cavity,hypo)	I,II,III,IVa, IVb	T1,T2,T3,T4,N0,N1,N2a,N2b,N2c, N3	RT	Pre&post treatment PET/CT	40%SUVmax

							<60y=30 ≥60y=26							
Lv et al (2019)(25)	Prediction of outcome	R	Hospital based(2012-2016	China	China	128 (TC-85,VC-43);M-103,F-25	Mean-47.7±13.2 Y	Median-23 (range 1-56m)	Naso	I,II,III,IV	T1,T2,T3,T4,O,N1,N2,N3,M0,M1	RT,CRT	PET/CT	Manual
Lv et al (2020)(26)	Prognostication in HNSCC	R	TCIA(2006-2014)	China	Canada	296, M=225,F=71	Median-CHUM-63(44-90)Y, CHUS-64(34-88)Y,HGJ-61(18-84)Y,HMR-67(49-85)Y	median 44(: 6–113)m	unknown, naso,oro,hypo,larynx	I,II,III,IV	Tx,T1, T2,T3,T4, N0,N1,N2, N3	RT,CRT	PET/CT	Manual
Lv et al (2021)(27)	Value of intra an peritumoural PET/CT in outcome prediction	R	TCIA(2006-2014)	China	Canada	166-M-125,F-41	CHUM-65±8, CHUS-66±9, HGJ-65±11,HMR-67±9	Median-43(6-112)m	Oro,hyp, naso, larynx,unknown	I,II,III,IV	T1,T2,T3,T4,Tx,N0,N1,N2,N3	RT, CRT	PET/CT	Manual
Martens et al (2020)(28)	Prediction of locoregional recurrence, DM, OS	R	Hospital based	Netherlands	Netherlands	103 retrospectively (training cohort) and 71 consecutively included patients (validation cohort) TC-M-76,F-27;VC-M-53,F-18	Mean TC- 62.3 (57.3–67.8) VC- 63.3 (57.8–69.3)	Mean-TC-31.5m(20.7-44.5), VC-26.4(19.8-34.1)	HNSCC (oro,hypo)	NR	T2,T3,T4,N0,N1,N2,N3	CRT	PET/CT	50% iso contour of SUVpeak

Oh et al (2015)(29)	Predict response to CRT and survival	R	Hospital based(2006-2011)	Republic of Korea	Republic of Korea?	70,M-64,F-6	Median-64 (range 40–73)Y	Median for survivors-56(18-100)m	Hypo	I,II,III,IV	T1,T2,T3,T4,N0,N1,N2,N3	Chemotherapy,RT,CRT	PET/CT	Manual
Peng et al (2019) (30)	Role of deep learning in risk stratification and treatment guidance	R	Hospital based(2009-2014)	China	China	707(TC-470, M-359,F-111,VC-237,M-175,F-62)	median-TC-45 (9–76)Y VC- 44 (10–76)Y	Median-55.7(1.3–93.6)m	Naso	III,IVa	T1,T2,T3,T4,N0,N1,N2,N3	CRT	PET/CT	Manual
Peng et al (2021)(31)	Predict locoregional recurrence and DM	R	Hospital based(2012-2016)	China	China	85;M-67,F-18	mean age 45 ± 12y, range 15–74 years	Median 25(range-2-54)m	Naso	III,IVb	T1,T2,T3,T4,N0,N1,N2,N3,N4	RT,CRT	PET/CT	Manual
Vallieres et al (2017)(32)	Predict Locoregional recurrence and DM	R	Hospital based(multiple hospital) (2006-2014)	Canada	Canada	298; (TC=194 VC=106) M-226,F-71	Mean HGJ- 61 ± 11, CHUS-64 ± 10, HMR- 67 ± 9, CHUM-63 ± 9	Median-43 (range: 6–112)mo	oro, hypo, naso, larynx, unknown)	I,II,III,IV, unknown	T1,T2,T3,T4, Tx,N0,N1,N2,N3	RT,CRT	PET/CT	Manual
Wang et al (2020)(33)	Prediction of Locoregional recurrence	R	TCIA(2006-2014)	USA	Canada	277	NR	43 (6-112) mo	NR	NR	NR	RT,CRT	PET/CT	Manual

Wong et al (2019)(34)	Evaluate clinical value of multimodality imaging parameters	R	Hospital based(2010-2013)	Taiwan	Taiwan?	61,M-59,F-2	Mean-51±10	Median-3y	hypo	III, IVa, IVb	T1,T2,T3,T4a,T4b,N0, N1,N2b,N2c	CRT,chemotherapy, RT	PET/CT	SUV threshold of 2.5
Xie et al (2020) (35)	Study the effect of resampling technique in the context of machine learning based model	R	Hospital based (2009-2016) & TCIA(2006-2014)	China	China & Canada	348(two different cohorts) Local cohort-166, M-123,F-43;tcia-182(M-144,F-38)	Mean Local-50.1y TCIA-62.9 y	Median Local- 79(2–131)m TCIA: 42(8–99)m	Local-Naso; TCIA-HNSCC oro, hypo, naso, larynx, unknown)	I,II,III,IV	TX,T1,T2,T3,T4,N0,N1, N2,N3	RT	PET/CT	Local cohort-40%SUVmax TCIA-Manual
Xu et al (2020)(36)	Investigating usefulness of radiomic features from intratumoural subregions	R	Hospital based	China	China	128(TC-85,VC-43);M-103,F-25	TC-47(15-78)y, vc-49(21-74)	Median-24±14m	Naso	I, II,III,IV	NR	RT and Chemotherapy	PET/CT	Manual
Yoon et al	Risk stratification	R	TC-TCIA(2	South Korea	TC-USA	TC-70 VC-49	Median - TC-60.5 (35–91)Y	Median-TC-	HNSCC (oro, oral	II,III,IV	T2,T3,T4,N0,N1,N2,N3	Surgery,CRT,RT,Chemotherapy	PET/CT	Nestle’s adaptive

(2021)(37)	for OS &DM		003-2013) VC-Hospital based(2007-2017)		VC-South Korea		VC-60.0 (34–88)Y	62mo,VC-52mo	cavity, naso, hypo glottis, maxillary sinus					thresholding
Zhong et al (2021)(38)	Prediction of early recurrence	R	Hospital based(2008-2017)	UK	UK	72 (TC=57 M-39, F-18, VC=15, M-11,F-4)	mean TC=61(41-77Y), VC-60 (43-74)Y	Median=26 (12-105) m	Hypo and Larynx	II,III,IV	T1,T2,T3,T4,N0,N1,N2,N3,N4,M2,M3,M4	RT,chemoradiotherapy	PET/CT	SUV>1.5 times liver SUVmean

Abbreviation: R=Retrospective cohort, TC=Training Cohort, VC=Validation Cohort, TCIA=The Cancer Imaging Archive, M=Male, F=Female, RT=Radiotherapy, CRT=Chemoradiotherapy, mo/m=months, y/yrs=years, oro=oropharyngeal, naso=nasopharyngeal, hypo=hypopharyngeal, ACM=All-Cause Mortality, OS=Overall Survival, DM=Distant Metastasis

Supplementary Table S5: Summary of models of included studies

Author; Year	Outcome	Features			Model parameters								Performance metrics		
		Etiologic factors	No of features	Type of features	Modelling method	Model with best performance	Dimensionality reduction method	Important features	Resampling method	Imbalanced class correction	Performance evaluation	Model Discrimination Metrics	Internal validation	External validation/(Train - test split)	Conclusion
Beichel et al (2019) (8)	DFS		22	PET quantitative features	Cox PH	NR	NR	MTV, Glycolysis Q2, RA (rim average)	NR	NR	NR	C Statistics	MTV-0.647 Glycolysis Q2-0.645 RA-0.634	NR	none of the features at baseline is deemed to be significant due to the number of features investigated combined with the limited dataset size.
Bogowicz et al (2017)(9)	Local control		596-CT & PET features	Shape, intensity, GLCM,NGTDM, GLSZM,wavelet	1.Multivariable Cox regression with backward election 2.LASSO 3.Random Forest	PCA+ Multivariable cox regression with backward selection	1.PCA 2. Pearson correlation 3. Average clustering 4. Mutual information method 5. Minimum redundancy maximum relevance method	**R**:PET-Spherical disproportion, , PET-$GLSZM_{SZLGE}$, CT-$GLSZM_{size\_zone\_entropy}$	5-fold cross validation, bootstrap	NR	External validation	CI	$CI_{PET/CT}$-0.77	CI_{CT}-0.73, CI_{PET}-0.71, $CI_{PET/CT}$-0.73	Multimodality radiomis combining PET &CT did not improve local tumour control modelling. CT and PET radiomics show potential to be a prognostic biomarker

															in HNC with equally good discriminative power.
Chan et al (2017)(10)	OS, RFS	NR	NR	SUV max, SUV mean, TLG, MTV, Age, sex, stage, T,N classification ,PET based, histogram, NGLCM, NGTDM	Cox regression model	NR	Univariate analysis	**OS**: Age, EBV DNA load, Uniformity **RFS**: Skewness	NR	NR	NR	NR	NR	NR	intratumor heterogeneity on 18F-FDG PET scans is associated with OS and RFS in patients with primary NPC. Specifically,
Cheng et al (2013)(11)	PFS,DSS, OS	HPV, tobacco use	NR	Age, HPV status, PET features, Histogram features,NGLCM(Normalised Gray Level Cooccurence Matrix	CoxPH	NR	NR	**PFS:** age, tumour TLG, $NGLCM_{uniformity}$ **DSS**: age, tumour TLG, $NGLCM_{uniformity}$ **OS**: TLG, $NGLCM_{uniformity}$, HPV positivity	NR	NR	NR	NR	NR	NR	Uni- formity (NGLCM)represents an independent prognostic predictor in patients with

				),NGTDM											
Cheng et al (2015)(12)	PFS,DSS	Tobacco, alcohol		HPV positivity, age, to-bacco, alcohol consumption, Tstage, N stage, AJCC stage, PET parameters, 22 rad features(GLSZM, GLRLM)	Cox PH	NR	Step forward	**PFS**: $GLSZM_{ZSN}$ (16 bins), $NGLCM_{Uniformity}$ **DSS**: $GLSZM_{ZSN}$ (16 bins), $NGLCM_{Uniformity}$	NR	NR	NR	NR	NR	NR	ZSNU identified as an independent predictor of PFS and DSS.
Cheng et al (2020)(13)	OS, Relapse Free Survival	Smoking		Shape, histogram, PET features, GLCM, GLRLM, GLSZM,smoking, treatment, WHO	CoxPH		Spearman's correlation, univariate, multivariate	**OS:** ECOG 2 or N2c − N3, Subgroup 3 PET pattern **PFS**: ECOG 2 or N2c − N3, Subgroup 3 PET pattern	Bootstrap, Recursive Partitioning Algorithm (RPA), 5 fold cross validation	NR	Train and test(from within the cohort)1.5:1) Internal validation	C statistics, sen, acc,	OS Cindex-0.83 Sen-0.741 Acc-0.853 RFS C index-0.78	NR	tumor SUVmax, discretized in- tensity entropy, and ECOG 2 or N2c-N3 status are independently associated with survival endpoints in patients with MSGC.

				histology, AJCC stage, ECOG performance , T,N stage											
Feliciani et al (2018)(14)	Local control / treatment failure,PFS,OS	NR	75-rad features	histogram, textural,age, sex, size, site of primary tumours, clinical stage, nodal involvement	CoxPH	NR	Univariate, multivariate , spearman,LASSO	**PFS**: Chemotherapy, $GLRLM_{LILRE}$<br>**OS**: Gender, Age	10 fold cross validation	NR	NR	C statistics	PFS<br>C index-0.76	NR	Models including imaging biomarkers were always su-<br>perior to those with only clinical variables, even ifonly in the case of PFS, we had a statistically significant differenc
Folkert et al (2017)(15)	DM, Local failure	Smoking, histology	24	Overall Stage, T,N class, smoking status, KPS(Karnofsky performance status) , age, sex, PET	Multiparametric logistic regression, For survival analysis- CoxPH-for ACM, Fine and Gray's proportional subhazaed model- Local Failure, DM	NR	Univariate analysis, multivariate analysis, forward feature selection	ACM: KPS, T stage<br>DM: KPS, T stage, N-stage<br>R: KPS	Leave one out cross validation, 5 fold cross validation ,Train and test(independent cohort)	NR	External validation	AUC, Sen, Spe	ACM<br>AUC-0.65,Sen-0.67,Spe-0.60<br>Local Failure<br>AUC-0.73, Sen.-).65, Spe-0.85<br>DM<br>AUC-0.66<br>Sen-0.62<br>Spe-0.66	ACM<br>AUC-0.60,Sen-0.58,Spe-0.62<br>Local Failure<br>AUC-0.68, Sen.-0.67, Spe-0.70<br>DM	LF model retained significance in an independent population whereas the models for ACM<br>and DM did not reach statistical significance , but resulted in

				featurs, hisogram, shape, biologically equivalent dose with alpha/beta=10 [BED 10],GLCM										AUC-0.65 Sen-0.64 Spe-0.80	reasonable predictive performance.
Fujima et al (2018)(16)	OS, PFS	NR	14	PET, shape, histogram, GLCMage, T stage, N-stage, treatment	Cox Regression	NR	Univariate analysis, multivariate	<u>PFS: GLCMHomogeneity, Sphericity</u>	NR	NR	NR	NR	NR	NR	The quantitative parameters of homogeneity and sphericity obtained by FDG-PET can be useful for the prediction of the PFS ofpharynx SCC patients, especially when used in combination.
Ger et al (2019)(17)	OS	NR	NR	Intensity,GLCM,GLRL	Cox PH	NR	Forward selection,LASSO	OS: CT(MODEL)-volume,	10 fold cross validation	NR	Train and separate test(from	AUC	NR	AUC =0.59	unable to demon-strate an

				M,NGTDM			;Bootstrap LASSO	GLCMGLNU, GLCMInverse_Difference_Norm			within the cohort) Internal ; hinted about external validation				improvement in prediction accuracy in a subset ofpatients with the same imaging protocol compared to a patient cohort with different imaging protocols.
Ghosh et al (2020)(18)	OS	NR	46	Age, sex, tumour site, stage, TNM class ,RT dose, smoking history, surgery, PET quantitative, GLCM, GLRLM, NGLDM,GLZLM	DT, RF, GBDT, CoxPH	GBDT	Pearson Correlation, scaling-minmaxscaler, gradient boost,hyper parameter optimisation	OS: Primary tumour site, MTV, GLCMCorrelation	5 fold cross validation	SMOTE,over sampling, Train and test (from within the cohort)	Internal validation	Balanced accuracy, sensitivity, specificity, true negative rate, precision, F1 score	Balanced accuracy-88.25%, Sen-96.5%,Spe-80%,Precision-93%, F1 score-94% AUC-0.99	NR	The accuracy of GBDT classifier is better than CoxPH
Guezennec et al	OS, RFS	NR	35	PET features,	Cox PH	NR	Pearson correlation	OS: MTV, GLCMCo	30 fold cross validation	NR	NR	NR	NR	NR	MTV and GLCM Correlation

(2019)(19)				GLCM,GLRLM, NGLDM, GLZLM,sex, age, tumour site, treatment, pathologic stage, T class			univariate analysis,	rrelation, Treatment							were independent prognostic factors of OS in patients with HNSCC
Haider et al (2020)(20)	PFS, OS	Smoking,HPV	1037 CT and PET	HPV, Stage, Radiomic-shape, intensity, txture	Random Survival Forest	Radiomics or radiomics+AJCC utperformed AJCC alone model	HierarchicalClustering, pearsonRF, RIDGE	NR	3 fold stratified cross-validation to	NR	Internal validation	C statistics	CI HPV+ PFS-0.62 ± 0.05 (p = 0.02)(PET/CT features) OS-0.63 ± 0.08 (p = 0.06)(PET features)	NR	radiomic analysis provide complementary value for prognostication and risk-stratification beyond AJCC staging.
Kimura et al (2021)(21)	DFS, OS	NR	14-textural	PET (SUVmax) GLCM, GLRLM, NGLDM, GLZLM,age , sex, primary tumor	Cox PH	NR	Backward stepwise selection, univariable, multivariable	OS: Entropy, Dissimilarity DFS: GLCMEntropy, GLZLMLZHGE, GLRLMSRE	NR	NR	NR	NR	NR	NR	Entropy is a statistically significant prognostic factor of both OS and DFS.

				location, T,N class, histologic differentiation, perineural and lymphovascular invasion, and resection margin											
Lafata et al (2021)(22)	RFS	NR	55 rad features	NR	CoxPH	NR	unsupervised data clustering algorithm; bi-clustering procedure and unsuper-vised data reduction	NR	NR	NR	NR	NR	NR	NR	pre-treatment radiomic expression was not associated with clinical outcome (Fig.
Lin et al (2020)(23)	OS, PFS	NR	NR	Histogram, NGLCM, NGTDM, PET (TLG, MTV, SUV mean,	CoxPH	NR	Univariate analysis,multivariate analysis, Forward step wise selection, spearmans correlation	**OS**: TLG-M, EBV-DNA titers **PFS**: SUVmax(M)	NR	NR	NR	NR	NR	NR	PET-derived texture parameters provide complementary prognostic information to EBV DNA titers.

				SUV max)											
Liu et al (2020)(24)	OS,DFS	Smoking	56	PET based, histogram, shape, GLCM, GLRLM,NGTDM, GLSZM, wavelet decomposition,BMI, age, T stage, N stage, AJCC stage, smoking history, histology grade, cancer site	CoxPH	NR	Pearson correlation, LASSO, univariate, multivariate analysis	**R**: $NGLDM_{Coarseness}$, SMTV **OS**: $NGLDM_{Coarseness}$, SMTV	Bootstrap, 15 fold cross validation	NR	NR	C statistics, nomogram, calibration curve	<u>OS</u> C index-0.77(95% CI-0.70-0.84) <u>DFS</u> C Index-0.77(95% CI-0.70-0.83)	NR	Combining clinicopathological characteristic with radiomic features of pe-treatment PET/CT may substantially improve prediction of OS & DFS
Lv et al (2019)(25)	PFS	NR	3276	Age, sex, AJCC stage, TNM class, EBV DNA,	Cox PH (clinical,PET,CT, clinical+PET, Clinical+CT, PET+CT,	Model with PET and/or CT with clincal	Univariate analysis, multivariate, spearman' correlation, forward step wise feature	**PFS:** M stage, Age, VCA-IgA, N stage, CT-GLSZM_LHH_GL	NR	NR	Train and test(from within the cohort) Internal validation	C statistics	0.71 to 0.76	0.62 to 0.75	Combining PET and/or CT features with clinical parameters showed improved outcome

				shape, histogram, immunoglobulin A antibodies against EBV viral capsid antigen(VCA-IgA), lymphocyte code(LYM), neutrophil count(NEUT), hemoglobin(HGB), platelet count(PLT), and lactate dehydrogenase level(LDH) PET & CT-	Clinical+PET+CT)	parameters	selection using maximum log likelihood	V_256(wavelet based), PET-SUVmid_HLH(wavelet based)							prediction relative to models with PET or CT radiomics features or clinical parameters alone in both training and validation cohorts

				GLCM, GLRLM,GLSZM, NGTDM, GLGLM, NGLDM,TS,TFC,TFCM											
Lv et al (2020) (26)	RFS,MFS, OS	NR	127-rad features	Clinical,PET parameters,shape, intensity,GLCM,GLRLM,GLSZM, NGTDM,GLGLM,NGLDM, GLDZM, moment features	CoxPH-different fusion models	RFS WF-0.6 MFS WF-0.8 OS no fusion model	NR	**RFS:** Age, $GLSZM_{SZHGE}$, $GLRLM_{LRHGE}$, B3 **OS**:NR	3 fold cross validation	NR	External validation and internal validation Train-test partition	C statistic		RFS WF0.6-C-index: 0.60± 0.04) MFS WF0.8 (0.71 ± 0.13) OS no fusion model significantly outperformed Clinical only, PET only or CT only models.	Fusion radiomics modeling showed varying improvements compared to single modal- ity models for different outcome predictions in different partitions, highlighting the importance of generalizing radiomics models

Lv, W et al (2021)(27)	OS,DM,LR	NR	6294	Clinical,Shape, first order, GLCM GLRLM GLSZM GLDM NGTDM	Ensemble Logistic regression(92 models)	LR Clinical+CT (Intra+Peri_6) DM PET(Intra+Peri_3) OS Clinical+PET_Peri_6	Combat,Gain equation, stepwise forward selection	NR	Bootstrap	Using ensemble of SVM	Train-111,Test-55(from within the cohort) and External validation	AUC, Sen,Spe, C statistics	NR	LR AUC-0.75, C-0.71,Spe-0.84,Sen-0.60; DM AUC-0.8; C-0.80; Sen-0.82, Spe-0.66 OS AUC-0.87; C-index - 0.83; Sen-0.88; Spe-0.61	Combat-in intratumoural PET model not obvious PET and CT radiomics features - provided additional infor-mation to clinical features. quantification of peri-tumoral micro-environment in PET and CT images integrated with clinical features can help to distinguish high risk patients who are easier to develop LR, DM or death in H&N cancer
Martens et al (2020)(28)	Locoregional recurrence ,DM	NR	434	Shape, histogram, GLCM, GLSZM, GLDZM, NGL	Cox regression(clinical parameter, PET parameter, radiomics parameter, combined clinical+PE	Cox regression with combined clinical+PET +radiomics	Redundancy filtering algorithm,,ridge regularisation,spearman's correlation, maximum likelihood	**R:** HPV-status, SUVmean , SUVpeak, histogram gradient,long-run-low-grey-level-	5 fold cross validation, Train and test(from same centre at different time point)	NR	External validation	C statistics	Recurrence CI-0.779(SE =0.050) Metastasis CI-0.657(SE =0.093), OS	Recurrence CI-0.645(SE=0.071) Metastasis CI-0.627(	Combining HPV-status, first-order 18F-FDG-PET parameters, and complementary radiomic factors was

				DM, NTGDM, NGLDTM, NGTDM	T parameter, combined clinical+radiomics, combined clinical+PET+radiomics)	features	factor analysis	emphasis, volume-difference, coarseness, and grey-level-non-uniformity and histogram variation coefficient **DM**: MATV **OS**: HPV-status, SUVmean, SUVmax, least-axis-length, non-uniformity, high-dependence-of-high grey-levels, asphericity, major-axis-length, inversed-compactness and, inversed-flatness					CI-0.751(SE=0.045)	SE=0.094), OS CI-0.764(SE=0.062)	most accurate for time-to-event prediction
Oh et al (2015)(29)	DFS,OS	Smoking,drinking,ECOG,Charson		SUV, MTV textural	CoxPH	NR	NR	**OS**: $NGTDM_{Coarsness}$ **DFS**: $NGTDM_{C}$	NR	NR	NR	NR	NR	NR	Abnormal coarseness in baseline 18F-FDG PET scans

		comorbidity index						oarsness, NGTDMBusyness							may be useful for predicting response and survival after CRT in HPSCC patients
Peng H et al (2019)(30)	DFS,OS, DMFS,LRRFS	Smoking, drinking, clinicopathological	136-deep learning,133-hand crafted	age, gender, smoking, drinking, family history ofcancer, lactate dehydrogenase, hemoglobin, albumin, C-reaction protein, T category, N category, and overall stage, pre-DNA shape	Cox proportional hazard(nomogram) PET based,CT based	NR	ICC, univariate analysis, pearsoncorrelaton, LASSO Cox regression method,Backward stepwise selection	NR	Train test split (70%:30%)	NR	Calibration curve; Internal	C statistics	Train(PET based) DFS-0.730 (95% CI, 0.683–0.776), Test DFS 0.683 (95% CI, 0.610–0.755)	NR	Deep learning PET/CT-based radiomics could serve as a reliable and powerful tool for prognosis prediction

				features, histogram features, GLCM,GLRLM											
Peng L et al (2021)(31)	Locoregional recurrence , DM (Disease control or treatment failure)	NR	127	histogram, shape, GLCM, GLRLM, GLSZM, NGTDM, GLGLM, NGLDM, TS, TFC ,age, sex, T, N and M stage, AJCC stage, pre-treatment plasma EBV DNA, immunoglobulin A	SVM, RF, Artificial Neural Network(ANN)	SFFS + SVM with LOOCV	Univariate analysis, multivariate analysis, relief algorithm, sequential floating forward selection(SFFS), hierarchical clustering, minimum redundancy maximum relevance	Compactness1, $GLCM_{Entropy}$, $GLSZM_{LZLGE}$, $NGTDM_{Strength}$, $GLGLM_{SGE}$	fivefold cross-validation (5 CV), tenfold cross-validation (10 CV) and leave-one-out cross-validation (LOOCV)	NR	Internal validation	AUC, Sen, spe	AUC-0.8290 Spe-0.7736 Sen-0.8438	NR	The SFFS feature selection coupled with SVM classifier can derive the optimized feature set with correspondingly highest AUC value for pretreatment prediction of LR and/or DM of NPC

				(IgA) antibodies against EBV viral capsid antigen, lymphocyte count (LYM), neutrophil count (NEUT), platelet count, lactate dehydrogenase level											
Vallières et al (2017)(32)	OS,DM, Locoregional recurrence		1615-rad features	PET features, age, tumour stage, Histogram, shape, GLCM, GLRLM, GLSZ	Random Forest(radiomics+clinical/ clinical alone), logistic regression(radiomics only/volume)), Cox PH(radiomics only/volume)	<u>Locoregional recurrence</u> - PETCT radiomics+clinical variables[LogReg+RF] <u>DM</u>	Stepwise forward selection, , spearman's correlation, maximal information coefficient, gain equation, logit transformation	***DM***- CT-$GLSZM_{ZSN}$, CT-$GLSZM_{ZSV}$, CT-$GLRLM_{LRHGE}$, H&N type, N-Stage Age, Tumour volume **R**-Age, CT-	0.632+bootstrap AUC	Stratified random sub sampling, imbalance adjustement mentioned in Schiller et	External validation	AUC, sen, spe, acc, C statistics	NR	LR AUC-0.69,sen-0.63,spe-0.68,acc-0.67,CI-0.67<br><br>DM AUC-0.86,sen-0.76,s	The combination of radi-omics data into clinically-integrated prediction models should allow to more comprehensively assess cancer risks

				M,NGTDM	Raiomics only(for PET,CT,PET-CT,)Tadiomics+clinical model(PET,CT,PET-CT)	CT adiomics+clinical variables[LogReg+RF]<br><br>OS Clinical variables alone[RF]		$GLSZM_{LGZE}$, PET-$GLSZM_{GLN}$, CT-$GLCM_{Correlation}$, H&N type, N-Stage, T stage<br>**OS**- H&N type,T stage,Age, Tumour volume, HPV		al, undue/over sampling, Train and test (from within the cohort)				pec-0.76, acc-0.77,CI-0.88<br><br>OS AUC-0.78,sen-0.92, spe-0.57, acc-0.65,CI-0.76	
Wang et al (2020) (33)	Locoregional Recurrence	NR	257-features	intensity, shape, and radiomic(GLCM)	SVM,DA, Logistic Regression ,MC	MC	mRMR,hyperparameter mentioned	NR	all experiments were performed five times	SMOTE;Oversampling	External validation	Sen,Spe, AUC,Acc.		MC with PET Features(compared to SVM, DA,Log Reg) AUC-0.62 ±0.03 Sen-0.62 ±0.12 Spe-0.61± 0.03 Acc-0.61 ±0.01	the model using features from multiple modalities, the proposed method achieved area under the receiver operating characteristic curve (AUC) values of 0.76 for the radio- mics-only model, and 0.77 for the model built with radiomics and clinical features, which is

															significantly higher than the AUCs of models built with single-modality features.
Wong et al (2019)(34)	RFS, OS	Smoking, alcohol, histology		Sex, stage, tobacco,alcohol,age, PET features, NGTDM, NGLCM, MRI variables	CoxPH	NR	Univariate analysis,multivariate analysis	**RFS**: transfer constant (Ktrans), TLG, $NGLCM_{entropy}$<br>**OS**: Ktrans, blood plasma volume (Vp), SUV_{max}, $NGLCM_{entropy}$	NR	NR	NR	NR	NR	NR	PET- and MRI-derived functional parameters may have a different prognostic significance in patients with oropharyngeal and hypopharyngeal carcinoma
Xie et al (2020)(35)	OS,DFS	NR	NR	Conventional, SUV max, SUV mean, MTV, TLG, GLCM, GLRLM, GLSZM	Logistic regression (LR), Support vector machine (SVM), random forest (RF), and XGboost classifier (XG)	NPC Cohort<br>DFS<br>bSMOTE + RF<br>OS<br>ADASYN + SVM<br>HNC cohort<br>DFS& OS<br>RF + SMOTE	ICC	NR	Monte carlo Cross validation;	Oversampling(random oversampling (ROS), adaptive synthetic (ADASYN) SMOTE	train test split(3:1) Internal; external validation mentioned.	AUC, G-mean	NPC Cohort<br>DFS<br>AUC-0.70,G-mean-0.64<br>OS<br>AUC-0.82, G-mean-0.77<br>HNC Cohort<br>DFS<br>AUC-0.72<br>OS<br>AUC-0.84	NR	re-sampling techniques showed a significant positive impact on the prediction performance in imbalanced datasets, but depending on the clinical problem and dataset, the performance of each

										and borderline-SMOTE (bSMOTE), undersampling(random undersampling (RUS), Near Miss Tomek link (TL) and edited dataset using nearest neighbours (ENN) ,I hybrid sampling-SMOTE-TL and					individual re-sampling techniques will vary.

										SMOTE-ENN imbalance rate mentioned					
Xu et al (2020)(36)	PFS	Pathology	202 - rad features	intensity, textural	Cox Regression	NR	Pearson correlation; Forward step wise feature selection, multivariate analysis	**PFS**: AJCC stage III-IV, CT-$GLGLM_{LGGE}$, PET-$NGTDM_{Complexity}$, PET-$GLGLM_{SGLGE}$	NR	NR	Train & Test (from within the cohort) Internal	C Statistics	C index S3-0.69	NR	Subregion imaging biomarker S3 was identified as an independent predictor of PFS and complementary to the existing AJCC staging system. Subregional radiomics analysis of PET/CT imaging has the potential to predict PFS in patients with NPC, which also provides complementary prognostic information for traditional predictors.

Yoon et al (2021)(37)	DFS, OS	NR	42-rad features	Age, sex, stage, PET, histogram, GLCM, GLRLM, NGLDM, GLZLM	Cox regression	NR	Filter based method, Univariate, pearson correlation, multivariater	**OS**: $GLZLM_{GLNU}$ **DFS**: $GLZLM_{GLNU}$	NR	NR	Train and validation (another cohort) External validation	NR	NR	NR	The meta-bolic heterogeneity parameter, GLNUGLZLM, may assist clinicians in patient risk assessment as a feasible prognostic factor.
Zhong et al (2021)(38)	PFS	Smoking	50-rad features	PET, shape, histogram, GLCM, NGLDM, GLRLM, GLZLM,duration of radiation treatment, N stage, smoking, age, sex	Random Forest (PET,CT, clinical, combined PET-CT)	Combined PET-CT radiomics model	Recursive Feature Elimination, dummy variable, hyperparameter-10cv	MTV, maximum CT value, SUVmin, GLZLMSZLGE, kurtosis	10-fold cross validation	Adjusted class weights	Training and validation (80%:20%) Internal validation	AUC, Acc, Sen, Spe,positive predictive value,f1score	AUC 0.93	Validation cohort AUC= 0.94. Acc-0.80 Sen=1 Spe-0.67 Positive Predictive value-1, F1 score= 0.77	A combined model encompassing PET and CT radiomic features had a higher AUC value and potentially had the best predictive ability for early disease progression (at 1 year) compared to individual PET, CT, and clinical feature models.

Abbreviation: PFS=Progression Free Survival, DFS=Disease Free Survival, DSS=Disease Specific Survival, Sen=Sensitivity, Spe= Specificity, Acc=Accuracy, NR=Not reported, SVM=Support Vector Machine, MC=Multi Classifier, DA=Discriminant Analysis

Supplementary Table S6: PROBAST assessment of Risk of Bias and applicability of included studies.

Study	ROB				Applicability			Overall	
	Participants	Predictors	Outcome	Analysis	Participants	Predictors	Outcome	ROB	Applicability
Beichel et al (2019)(8)	+	+	+	-	?	+	+	-	?
Bogowicz et al (2017)(9)	+	+	+	?	+	+	+	?	+
Chan et al (2017)(10)	+	+	+	?	+	?	?	-	?
Cheng et al (2013)(11)	+	+	?	?	?	?	?	-	?
Cheng et al (2015)(12)	+	+	+	?	?	+	?	-	?
Cheng et al (2020)(13)	+	+	+	?	?	?	+	-	?
Feliciani et al (2018)(14)	+	+	+	?	+	+	?	-	?
Folkert et al (2017)(15)	+	+	+	?	?	+	?	?	?
Fujima et al (2018)(16)	+	?	+	-	?	+	+	-	?
Ger et al (2019)(17)	+	+	+	?	+	+	+	?	+
Ghosh et al (2020)(18)	+	+	?	?	+	?	?	-	?
Guezennec et al (2019)(19)	+	+	+	?	+	?	+	-	?
Haider et al (2020)(20)	+	+	+	+	+	+	+	-	+
Kimura et al (2021)(21)	+	+	+	?	?	+	+	-	?
Lafata et al (2021)(22)	+	+	+	-	?	+	+	-	?
Lin et al (2020)(23)	+	+	+	-	-	?	+	-	-
Liu et al (2020)(24)	+	+	?	?	+	+	?	-	?
Lv et al (2019)(25)	?	+	+	-	+	?	+	-	?

Lv et al (2020)(26)	+	+	+	+	+	+	+	+	+
Lv et al (2021)(27)	+	+	+	+	+	+	+	+	+
Martens et al (2020)(28)	+	+	+	+	+	+	+	+	+
Oh et al (2015)(29)	+	+	+	-	?	+	+	-	?
Peng et al (2019)(30)	+	+	+	?	+	?	+	-	?
Peng et al (2021)(31)	?	+	?	?	?	+	+	-	?
Vallières et al (2017)(32)	+	+	+	+	+	+	+	+	+
Wang et al (2020)(33)	+	+	+	+	+	?	?	+	?
Wong et al (2019)(34)	+	+	+	-	?	+	+	-	?
Xie et al (2020)(35)	+	?	+	?	+	+	+	?	+
Xu et al (2020)(36)	+	+	-	-	+	+	?	-	?
Yoon et al (2021)(37)	+	?	+	?	?	?	+	?	?
Zhong et al (2021)(38)	+	+	?	+	?	+	+	-	?

+ indicates low ROB/low concern regarding applicability; − indicates high ROB/high concern regarding applicability; and ? indicates unclear ROB/unclear concern regarding applicability.

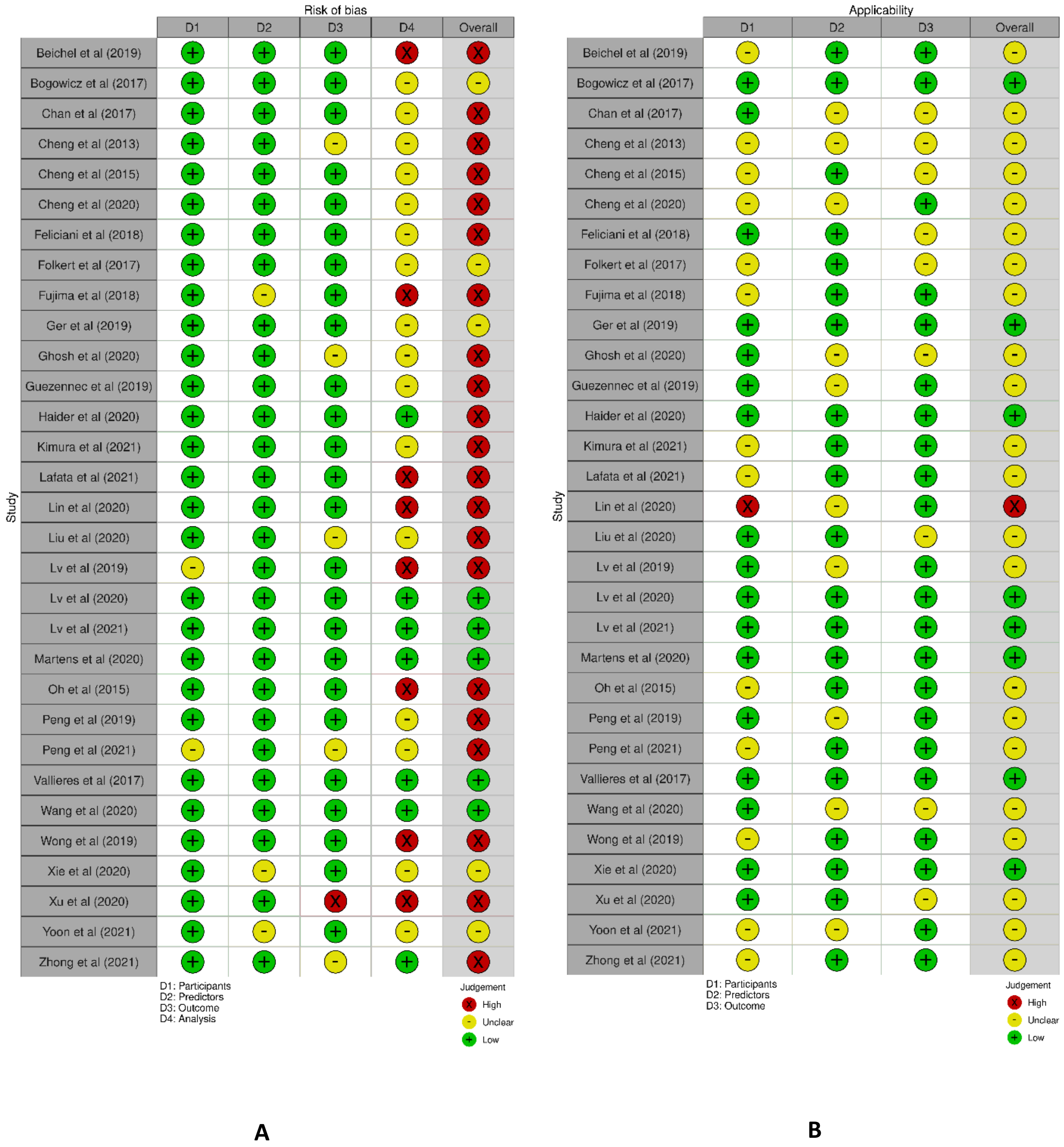

Fig. S1 Quality analysis of the included studies based on PROBAST

(A) Overall Risk of Bias (ROB) and (B) Overall applicability.

Green represents low ROB/low concern, yellow represents unclear ROB/ unclear concern, and red represents high ROB/high concern criteria(39)

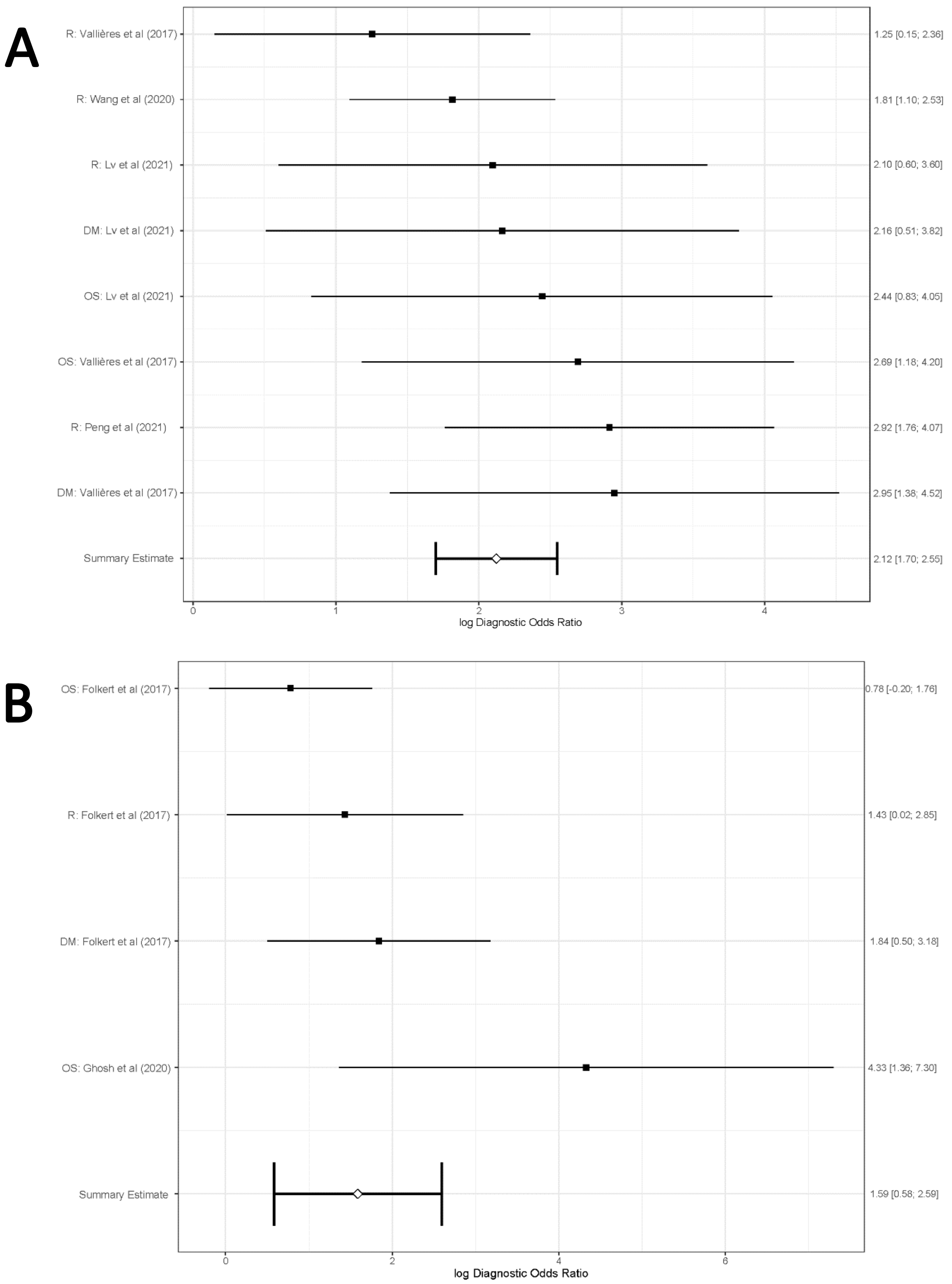

Fig. S2 Forest plots of prediction models on subset of studies

Forest plot of the summary estimate of logarithmic DOR and the corresponding 95% confidence interval (CI) of prediction models in which images were segmented using (A) Manual method and (B) thresholding-based segmentation method. Performance metrics were based on external validation except for Ghosh et al (2020) and Peng et al (2021), where the performance metrics were based on internal validation.

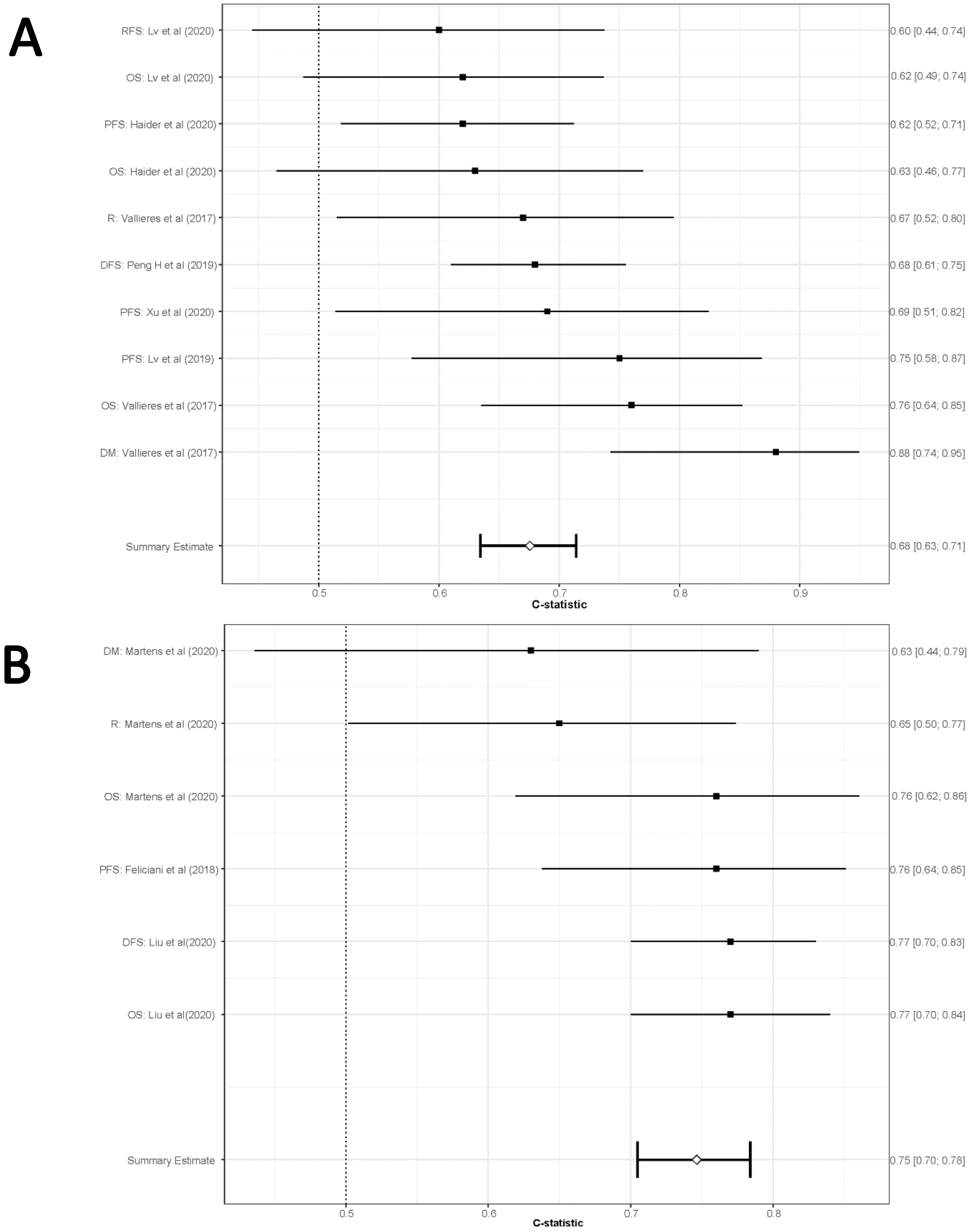

Fig. S3 Forest plots of prognostic models on subset of studies

Forest plot of the summary estimate of logarithmic DOR and the corresponding 95% confidence interval (CI) of prediction models in which images were segmented using (A) Manual method and (B) thresholding-based segmentation method. Performance metrics were based on internal validation except for Lv et al (2020), Martens et al (2020) and Vallières et al (2017), where the performance metrics were based on external validation.

References

1. Mendeley Reference Manager | Mendeley [Internet]. [cited 2022 Jun 23]. Available from: https://www.mendeley.com/reference-management/reference-manager

2. Moons KGM, De Groot JAH, Bouwmeester W, Vergouwe Y, Mallett S, Altman DG, et al. Guidelines and Guidance Critical Appraisal and Data Extraction for Systematic Reviews of Prediction Modelling Studies: The CHARMS Checklist. Available from: www.plosmedicine.org

3. Moons KGM, Wolff RF, Riley RD, Whiting PF, Westwood M, Collins GS, et al. PROBAST: A tool to assess risk of bias and applicability of prediction model studies: Explanation and elaboration. Ann Intern Med [Internet]. 2019 Jan 1 [cited 2021 Jul 14];170(1):W1–33. Available from: www.probast.org

4. DerSimonian R, Laird N. Meta-analysis in clinical trials. Control Clin Trials. 1986 Sep 1;7(3):177–88.

5. Knapp G, Hartung J. Improved tests for a random effects meta-regression with a single covariate. Stat Med. 2003 Sep 15;22(17):2693–710.

6. Doebler P, Holling H. Meta-Analysis of Diagnostic Accuracy with mada. [cited 2022 Jul 8]; Available from: http://r-forge.r-project.org/projects/mada/;

7. Debray TPA, Jong V de. 'metamisc' Meta-Analysis of Diagnosis and Prognosis Research Studies. 2021 [cited 2022 Jul 8]; Available from: https://orcid.org/0000-0002-1790-2719

8. Beichel RR, Ulrich EJ, Smith BJ, Bauer C, Brown B, Casavant T, et al. FDG PET based prediction of response in head and neck cancer treatment: Assessment of new quantitative imaging features. PLoS One [Internet]. 2019 Apr 1 [cited 2021 Apr 20];14(4):e0215465. Available from: https://doi.org/10.1371/journal.pone.0215465

9. Bogowicz M, Riesterer O, Stark LS, Studer G, Unkelbach J, Guckenberger M, et al. Comparison of PET and CT radiomics for prediction of local tumor control in head and neck squamous cell carcinoma. Acta Oncol (Madr) [Internet]. 2017 Nov 2 [cited 2021 Apr 20];56(11):1531–6. Available from: http://ovidsp.ovid.com/ovidweb.cgi?T=JS&PAGE=reference&D=med14&NEWS=N&AN=28820287

10. Chan S-C, Chang K-P, Fang Y-HD, Tsang N-M, Ng S-H, Hsu C-L, et al. Tumor heterogeneity measured on F-18 fluorodeoxyglucose positron emission tomography/computed tomography combined with plasma Epstein-Barr Virus load predicts prognosis in patients with primary nasopharyngeal carcinoma. Laryngoscope [Internet]. 2017;127(1):E22–8. Available from: http://ovidsp.ovid.com/ovidweb.cgi?T=JS&PAGE=reference&D=med14&NEWS=N&AN=27435352

11. Cheng NM, Fang YHD, Chang JTC, Huang CG, Tsan DL, Ng SH, et al. Textural features of pretreatment 18F-FDG PET/CT images: Prognostic significance in patients with advanced T-stage oropharyngeal squamous cell carcinoma. J Nucl Med. 2013;54(10):1703–9.

12. Cheng NM, Fang YHD, Lee LY, Chang JTC, Tsan DL, Ng SH, et al. Zone-size nonuniformity of F-18-FDG PET regional textural features predicts survival in patients with oropharyngeal cancer. Eur J Nucl Med Mol Imaging. 2015;42(3):419–28.

13. Cheng N-MM, Hsieh C-EE, Fang Y-HHD, Liao C-TT, Ng S-HH, Wang H-MM, et al. Development and validation of a prognostic model incorporating [18F]FDG PET/CT radiomics for patients with minor salivary gland carcinoma. EJNMMI Res [Internet]. 2020;10(1):74. Available from: http://www.springerlink.com/content/2191-219x/

14. Feliciani G, Fioroni F, Grassi E, Bertolini M, Rosca A, Timon G, et al. Radiomic profiling of head and neck cancer: 18F-FDG PET texture analysis as predictor of patient survival. Contrast Media Mol Imaging [Internet]. 2018;2018:3574310. Available from: https://www.hindawi.com/journals/cmmi/contents/

15. Folkert MR, Setton J, Apte AP, Grkovski M, Young RJ, Schöder H, et al. Predictive modeling of outcomes following definitive chemoradiotherapy for oropharyngeal cancer based on FDG-PET image characteristics. Phys Med Biol [Internet]. 2017 Jun 12 [cited 2021 Feb 8];62(13):5327–43. Available from: https://pubmed.ncbi.nlm.nih.gov/28604368/

16. Fujima N, Hirata K, Shiga T, Li R, Yasuda K, Onimaru R, et al. Integrating quantitative morphological and intratumoural textural characteristics in FDG-PET for the prediction of prognosis in pharynx squamous cell carcinoma patients. Clin Radiol [Internet]. 2018 Dec 1 [cited 2021 Mar 9];73(12):1059.e1-1059.e8. Available from: http://ovidsp.ovid.com/ovidweb.cgi?T=JS&PAGE=reference&D=med15&NEWS=N&AN=30245069

17. Ger RB, Zhou S, Elgohari B, Elhalawani H, Mackin DM, Meier JG, et al. Radiomics features of the primary tumor fail to improve prediction of overall survival in large cohorts of CT- And PET-imaged head and neck cancer patients. PLoS One [Internet]. 2019;14(9):1–13. Available from: http://dx.doi.org/10.1371/journal.pone.0222509

18. Ghosh S, Maulik S, Chatterjee S, Mallick I, Chakravorty N, Mukherjee J. Prediction of survival outcome based on clinical features and pretreatment 18FDG-PET/CT for HNSCC patients. Comput Methods Programs Biomed. 2020;195.

19. Guezennec C, Robin P, Orlhac F, Bourhis D, Delcroix O, Gobel Y, et al. Prognostic value of textural indices extracted from pretherapeutic 18-F FDG-PET/CT in head and neck squamous cell carcinoma. Head Neck. 2019 Feb 1;41(2):495–502.

20. Haider SP, Mahajan A, Payabvash S, Haider SP, Baumeister P, Reichel C, et al. Potential added value of PET/CT radiomics for survival prognostication beyond AJCC 8th edition staging in oropharyngeal squamous cell carcinoma. Cancers (Basel) [Internet]. 2020 Jul 1 [cited 2021 Feb 12];12(7):1–16. Available from: https://pubmed.ncbi.nlm.nih.gov/32635216/

21. Kimura M, Kato I, Ishibashi K, Sone Y, Nagao T, Umemura M. Texture Analysis Using Preoperative Positron Emission Tomography Images May Predict the Prognosis of Patients With Resectable Oral Squamous Cell Carcinoma. J Oral Maxillofac Surg [Internet]. 2021;79(5):1168–76. Available from: https://doi.org/10.1016/j.joms.2020.12.014

22. Lafata KJ, Yoo DS, Lafata KJ, Chang Y, Wang C, Yin F-F, et al. Intrinsic radiomic expression patterns after 20 Gy demonstrate early metabolic response of oropharyngeal cancers. Med Phys [Internet]. 2021;48(7):3767–77. Available from: http://aapm.onlinelibrary.wiley.com/hub/journal/10.1002/(ISSN)2473-4209/issues/

23. Lin H-CC, Chan S-CC, Cheng N-MM, Liao C-TT, Hsu C-LL, Wang H-MM, et al. Pretreatment F-18-FDG PET/CT texture parameters provide complementary information to Epstein-Barr virus DNA titers in patients with metastatic nasopharyngeal carcinoma. ORAL Oncol [Internet]. 2020;104:104628. Available from: http://ovidsp.ovid.com/ovidweb.cgi?T=JS&PAGE=reference&D=med18&NEWS=N&AN=32163890

24. Liu Z, Cao Y, Diao W, Cheng Y, Jia Z, Peng X. Radiomics-based prediction of survival in patients with head and neck squamous cell carcinoma based on pre- and post-treatment F-18-PET/CT. Aging (Albany NY) [Internet]. 2020 Jul 31 [cited 2021 Feb 12];12(14):14593–619. Available from: https://pubmed.ncbi.nlm.nih.gov/32674074/

25. Lv W, Yuan Q, Wang Q, Ma J, Feng Q, Chen W, et al. Radiomics Analysis of PET and CT Components of PET/CT Imaging Integrated with Clinical Parameters: Application to Prognosis for Nasopharyngeal Carcinoma. Mol Imaging Biol. 2019 Oct 1;21(5):954–64.

26. Lv WB, Ashrafinia S, Ma JH, Lu LJ, Rahmim A, Lv WB, et al. Multi-Level Multi-Modality Fusion Radiomics: Application to PET and CT Imaging for Prognostication of Head and Neck Cancer. IEEE J Biomed Heal Informatics [Internet]. 2020;24(8):2268–77. Available from: http://ieeexplore.ieee.org/xpl/RecentIssue.jsp?punumber=6221020

27. Lv WB, Feng H, Du DY, Ma JH, Lu LJ. Complementary Value of Intra- and Peri-Tumoral PET/CT Radiomics for Outcome Prediction in Head and Neck Cancer. IEEE ACCESS. 2021;9:81818–27.

28. Martens RM, Koopman T, Noij DP, Pfaehler E, Übelhör C, Sharma S, et al. Predictive value of quantitative 18F-FDG-PET radiomics analysis in patients with head and neck squamous cell carcinoma. EJNMMI Res [Internet]. 2020 [cited 2021 Apr 20];10(1):102. Available from: http://www.springerlink.com/content/2191-219x/

29. Oh JS, Kang BC, Roh JL, Kim JS, Cho KJ, Lee SW, et al. Intratumor Textural Heterogeneity on Pretreatment F-18-FDG PET Images Predicts Response and Survival After Chemoradiotherapy for

Hypopharyngeal Cancer. Ann Surg Oncol. 2015;22(8):2746–54.

30. Peng H, Tang L-L, Chen L, Li W-F, Mao Y-P, Sun Y, et al. Prognostic Value of Deep Learning PET/CT-Based Radiomics: Potential Role for Future Individual Induction Chemotherapy in Advanced Nasopharyngeal Carcinoma. Clin Cancer Res [Internet]. 2019;25(14):4271–9. Available from: http://ovidsp.ovid.com/ovidweb.cgi?T=JS&PAGE=reference&D=med16&NEWS=N&AN=30975664

31. Peng L, Hong X, Yuan Q, Lu L, Wang Q, Chen W, et al. Prediction of local recurrence and distant metastasis using radiomics analysis of pretreatment nasopharyngeal [18F]FDG PET/CT images. Ann Nucl Med [Internet]. 2021 Apr 1 [cited 2021 May 25];35(4):458–68. Available from: http://www.jsnm.org/paper2/index_anm_English.htm

32. Vallières M, Kay-Rivest E, Perrin LJ, Liem X, Furstoss C, Aerts HJWL, et al. Radiomics strategies for risk assessment of tumour failure in head-and-neck cancer. Sci Rep [Internet]. 2017 Dec 1 [cited 2021 Feb 12];7(1). Available from: https://pubmed.ncbi.nlm.nih.gov/28860628/

33. Wang K, Chen L, Sher D, Wang J, Zhou Z, Wang R, et al. A multi-objective radiomics model for the prediction of locoregional recurrence in head and neck squamous cell cancer. Med Phys [Internet]. 2020 Oct 1 [cited 2021 Apr 20];47(10):5392–400. Available from: https://pubmed.ncbi.nlm.nih.gov/32657426/

34. Wong C-K, Chan S-C, Ng S-H, Hsieh C-H, Cheng N-M, Yen T-C, et al. Textural features on 18F-FDG PET/CT and dynamic contrast-enhanced MR imaging for predicting treatment response and survival of patients with hypopharyngeal carcinoma. Medicine (Baltimore) [Internet]. 2019;98(33):e16608. Available from: http://ovidsp.ovid.com/ovidweb.cgi?T=JS&PAGE=reference&D=med16&NEWS=N&AN=31415354

35. Xie CY, Du R, Chiu KWH, Lee EYP, Vardhanabhuti V, Ho JWK, et al. Effect of machine learning re-sampling techniques for imbalanced datasets in F-18-FDG PET-based radiomics model on prognostication performance in cohorts of head and neck cancer patients. Eur J Nucl Med Mol Imaging [Internet]. 2020;47(12):2826–35. Available from: http://ovidsp.ovid.com/ovidweb.cgi?T=JS&PAGE=reference&D=med18&NEWS=N&AN=32253486

36. Xu H, Lv W, Feng H, Du D, Yuan Q, Wang Q, et al. Subregional Radiomics Analysis of PET/CT Imaging with Intratumor Partitioning: Application to Prognosis for Nasopharyngeal Carcinoma. Mol Imaging Biol. 2020 Oct 1;22(5):1414–26.

37. Yoon H, Ha S, Jin Kwon S, Youngju Park S, Kim J, Hyun JO, et al. Prognostic value of tumor metabolic imaging phenotype by FDG PET radiomics in HNSCC. Ann Nucl Med. 2021 Mar;35(3):370–7.

38. Zhong J, Frood R, Brown P, Nelstrop H, Prestwich R, McDermott G, et al. Machine learning-based FDG PET-CT radiomics for outcome prediction in larynx and hypopharynx squamous cell carcinoma. Clin Radiol [Internet]. 2021 Jan 1 [cited 2021 May 25];76(1):78. Available from: http://www.elsevier.com/inca/publications/store/6/2/3/0/1/9/index.htt

39. robvis [Internet]. [cited 2023 Mar 10]. Available from: https://mcguinlu.shinyapps.io/robvis/
